# Introgression of barley chromosome arms 4H and 6H into wheat via Robertsonian translocations: GBS-assisted structural analysis and impact on grain nutrient composition

**DOI:** 10.1007/s11103-026-01689-8

**Published:** 2026-02-18

**Authors:** László Ivanizs, Eszter Gaál, Klaudia Kruppa, András Farkas, Péter Mikó, Edina Türkösi, Marianna Rakszegi, Péter Kovács, Balázs Kalapos, Andrea Gulyás, Norbert Hidvégi, Kitti Szőke-Pázsi, Márta Molnár-Láng, Éva Szakács, Mahmoud Said, Jan Bartoš, Tünde Pusztahelyi, Dimitar Douchkov, István Molnár

**Affiliations:** 1 HUN-REN Centre for Agricultural Research, Agricultural Institute, Martonvásár, Hungary; 2 Centre of Plant Structural and Functional Genomics, Institute for Experimental Botany, Olomouc, Czech Republic; 3 Agricultural Research Centre, Field Crops Research Institute, Giza, Egypt; 4 Central Laboratory of Agricultural and Food Products, Faculty of Agricultural and Food Sciences and Environmental Management, University of Debrecen, Debrecen, Hungary; 5 Leibniz Institute of Plant Genetics and Crop Plant Research (IPK), OT Gatersleben, Seeland, Germany

**Keywords:** Wheat-barley translocation, *In situ* hybridization, Genotyping-by-sequencing, Read coverage analysis, Chromosome-mediated gene transfer, Mineral components

## Abstract

In addition to wild relatives, cultivated *Triticeae* species such as barley can also serve as an excellent gene source to improve the agronomic traits of bread wheat. The Asakaze-Manas addition lines previously developed in Martonvásár exhibit low fertility and unstable inheritance; nonetheless, they could serve as ’bridge materials’ for the incorporation of barley chromatin into the wheat genome. By leveraging the centric breakage-fusion mechanism of unpaired chromosomes and the gametocidal effect of chromosome 2C from *Aegilops cylindrica*, the 4H and 6H addition lines were used to develop genetically stable translocation genotypes. In situ hybridization and molecular marker analyses were applied to identify the wheat and barley chromosome arms in the compensatory translocations, designated as T6HS.6BL, T6BS.6HL and T4BS.4HL. These results were confirmed by GBS read coverage analysis, which revealed a putative pericentric inversion in the barley chromosome arms involved in the T6HS.6BL and T6BS.6HL. The comparable agronomic performance of the centric fusion lines indicates that these chromosomal rearrangements had minimal, if any, negative impact relative to the parental wheat varieties. The newly developed translocation lines had an increased amount of minerals such as iron, suggesting that they could serve as promising genetic material for the biofortification of hexaploid wheat.

**Supplementary Information:**

The online version contains supplementary material available at 10.1007/s11103-026-01689-8.

## Key message

Molecular marker analysis and in situ hybridization techniques, combined with genotyping-by-sequencing, enable the selection of stable wheat-barley translocation lines, leading to chromosome-mediated improvement in grain quality.

**Supplementary Information:**

The online version contains supplementary material available at 10.1007/s11103-026-01689-8.

## Introduction

Allohexaploid wheat (*Triticum aestivum* L., 2n = 6x = 42; BBAADD) is a globally cultivated cereal, that plays a central role in human nutrition. Although thousands of years of breeding have resulted in higher yields of modern wheat varieties, this process has led to a narrowing of its genetic variability (Tiwari et al. 2014). The genetic potential of bread wheat can be enhanced via hybridization with its related species, which is an efficient way for chromosome-mediated transfer of agronomically useful genes. Barley (*Hordeum vulgare* L., 2n = 2x = 14; HH) as a cultivated relative of hexaploid wheat has proven to be a promising crossing partner for breeding programmes, whose gene pool represents a rich source of valuable traits such as earliness (Farkas et al. 2014) or high edible fibre (β-glucan) content (Cseh et al. 2013; Türkösi et al. 2018). The majority of barley varieties are resistant to Fusarium head blight (Huang et al. 2018) or wheat powdery mildew (Lück et al. 2025) and exhibit tolerance to osmotic and salinity stress (Darko et al. 2015, 2017), which can mitigate yield losses due to the negative impact of climate change.

Besides the adaptability and yield stability, a healthy diet has become an essential goal of cereal breeding programs in the 21st century. Barley is rich in essential amino acids (Åssveen 2009; Zijstra and Beltranena 2015; Huang et al. 2020), which are found in limited amounts in the wheat grain. Some studies revealed a wide variation of their composition in the smaller and larger collections of barley cultivars (Knežević et al. 2007; Huang et al. 2020). In addition, a significant difference was observed in microelement concentration (especially Fe) between wheat (15–22 mg/kg) and barley (24–79 mg/kg) (Bityutskii et al. 2017), with iron deficiency being a widespread nutritional problem affecting the diet of one-third of the human population (Hotz and Brown 2004). Therefore, due to its excellent nutritional value, barley can be utilized to improve the protein- and mineral composition of bread wheat, facilitating the production of functional foods. The grain protein content (GPC) in wheat is significantly influenced by *NAM-B1* (*TtNAM-B1*) gene mapped on the short arm of chromosome 6B (Uauy et al. 2006). The functional *TtNAM-B1* allele seems to have a pleiotropic effect, speeding up senescence of whole-plant and enhancing the remobilization of nitrogen and micronutrients from vegetative tissues to developing grains (Uauy et al. 2006). Distelfeld et al. (2008) dissected the collinearity between the wheat and barley genomic regions determining GPC, as a result, an orthologue of *TtNAM-B1* locus (*HvNAM-1*) was identified on the barley 6HS chromosome arm. Several candidate genes were detected and localised on chromosomes 4H and 6H that have a strong effect on the iron and zinc content in barley grain (Gyawali et al. 2017; Nyiraguhirwa et al. 2022). Incorporation of these chromosome arms into the wheat genome allows for information about how the transferred barley genes modify the protein composition and mineral concentration in bread wheat.

The Ukrainian six-rowed barley cultivar Manas, adapted to the climatic conditions of Central Europe, exhibits outstanding agronomic performance (yield and nutritional parameters), thereby representing an attractive gene source for wheat breeding. To introduce these new allelic variations into wheat, the winter barley ’Manas’ and Japanese facultative wheat ’Asakaze’ were crossed to develop hybrids (Molnár-Láng et al. 2000), then a series of addition lines containing special chromosomes from barley in the wheat genetic background. A partial set of the Asakaze-Manas addition lines (2H, 3H, 4H, 6H, 7H) produced previously in Martonvásár are suitable for studying the effect of barley chromosomes transferred into wheat (Molnár-Láng et al. 2012). Nevertheless, wheat-barley aneuploids have a certain level of karyotypic instability, since barley chromosomes have a tendency to be eliminated from the wheat background over generations (Szakács and Molnár-Láng 2010; Molnár-Láng et al. 2012). Therefore, maintenance of wheat-barley addition lines requires continuous cytogenetic monitoring, a time-consuming and labour-intensive process. Development of translocation lines with 42 chromosomes offers an opportunity to create genetically stable wheat pre-breeding materials carrying barley gene variants (Molnár-Láng et al. 2014).

One chromosome engineering strategy utilizes a special condition, where wheat and alien chromosomes are monosomic to induce centric breakage and fusion in the meiosis (Lukaszewski 1997, 2010; Friebe et al. 2005). Crossing a wheat line monosomic for a wheat chromosome with a wheat-barley disomic addition carrying the corresponding barley chromosome may result in F_1_ hybrid progenies containing wheat and barley univalents for the same homoeologous group in addition to 40 wheat chromosomes. In anaphase of meiosis I, the unpaired univalents may suffer breakage at their centromeres, after which the broken arms of wheat and barley chromosomes can occasionally fuse, forming Robertsonian translocations (Friebe et al. 2005). There have already been a few reports on non-homeologous (Cseh et al. 2011) and compensating (Danilova et al. 2018; Türkösi et al. 2018) Robertsonian translocations obtained from the misdivison-fusion mechanism between the monosomic chromosomes of wheat and barley.

The use of the gametocidal (*Gc*) system is another non-recombination mechanism to induce random translocations between wheat and alien chromosomes, including barley (Said et al. 2024). The gametocidal genes in *Aegilops* species can generate random chromosome breaks, resulting in various abnormalities such as deletions, insertions, inversions, or translocations (Endo 1990). Gametes containing *Gc* chromosomes are passed continuously to the next generation, whereas gametes lacking them are either aborted or undergo non-lethal chromosome breakages. The broken ends can join with other chromosome fragments, leading to the formation of translocations. Therefore, *Gc* genes can be used in chromosome-mediated gene transfer between different species (namely, wheat and barley). *Gc* chromosomes originating from various *Aegilops* species, including *Ae. cylindrica*, have been incorporated into the genetic background of wheat, producing addition lines (Endo 1990; Friebe et al. 1999; Kwiatek et al. 2017). Among these, the 2C chromosome of *Aegilops cylindrica*, the 3C of *Aegilops triuncialis*, and the 4M of *Aegilops geniculata* have been most frequently used as crossing partners with other wheat-alien addition lines to induce wheat-alien chromosome rearrangements, including wheat-barley introgressions (Schubert et al. 1998; Shi and Endo 2000). Numerous studies have reported that the gametocidal effect of the *Aegilops* chromosomes (2C, 3C) induced structural rearrangements in chromosomes 2H (Joshi et al. 2011), 3H (Sakai et al. 2009), 4H (Sakata et al. 2010), 5H (Ashida et al. 2007), 6H (Ishihara et al. 2014) and 7H (Serizawa et al. 2001) of the barley cultivar ’Betzes’ added to the wheat variety ’Chinese Spring’ (CS). Dissection lines containing rearranged barley chromosomes were developed from crosses of wheat-barley and wheat-*Aegilops* addition lines, which were used to physically map a number of barley-specific expressed sequence tags (ESTs).

The success of chromosome-mediated gene transfer depends on the ability to identify even minor alien chromosome segments in the wheat genetic background within a large population. It is necessary to use a high-capacity selection system that allows the detection of transferred chromatin at high resolution. Molecular cytogenetic methods, fluorescence- and genomic in situ hybridisation (FISH and GISH) are suitable techniques for identifying and tracking foreign chromosome fragments in wheat background, but they are less efficient for screening large populations (Kuraparthy et al. 2009). PCR-based molecular marker techniques, such as microsatellite or S-SAP markers, have opened the way for high-throughput selection in pre-breeding population (Nagy et al. 2002, 2006; Cseh et al. 2011; Türkösi et al. 2018). However, the physical position of marker sequences on the chromosome can only be localised with limited fidelity, making it difficult to utilize them for accurate mapping of the transferred chromatin segments. The combination of molecular marker analysis and in situ hybridization has been effectively applied worldwide to characterise wheat-barley introgressions, despite its limited resolution for detecting the small chromatin fragments (Rey et al. 2015).

Genotyping-by-sequencing (GBS) is a high-resolution multiplex method that allows the generation of large amounts of sequences covering the entire genome of a large number of individuals (Poland et al. 2012a; Pootakham et al. 2016). Hexaploid wheat has a large and complex genome with a huge amount of repetitive elements, which can be resolved by a reduced-representation sequencing approach through the use of two restriction endonucleases (frequent- and rare-cutter). The combination of double digestion of genomic DNA and next-generation sequencing (NGS) technologies offers the possibility to obtain high-throughput polymorphic short-reads spread over the whole genome, most of which originate from the gene-rich regions (Elshire et al. 2011). The advantages of GBS have been successfully exploited for genotyping, diversity analysis, association, linkage, and physical mapping of wheatgrasses (Comadran et al. 2011; Kumar et al. 2015; Baloch et al. 2017; Monostori et al. 2017; Dracatos et al. 2019). The extensive set of GBS markers provides a dense coverage of the whole genome; therefore, it is effectively applicable for detecting major and minor chromosome rearrangements in wheat breeding populations (Adhikari et al. 2022; Gaál et al. 2024; Kruppa et al. 2025).

The current work reports the development of two compensating wheat-barley translocation lines containing short (T6HS.6BL) or long arm (T6BS.6HL) of barley chromosome 6H, which were induced by the centric fission-fusion mechanism of the unpaired chromosomes. In addition, another homoeologous Robertsonian translocation encompassing the long arm (T4BS.4HL) of barley chromosome 4H is also presented here, which was produced using the gametocidal effect of chromosome 2C of *Ae. cylindrica*. The chromosome constitution of the newly developed translocation lines was examined using molecular cytogenetic methods (GISH and FISH). GBS read coverage analysis enables the detailed characterization and revelation of the chromosome constitution of centric fusions and wheat genetic background, by aligning with reference genomes of hexaploid wheat cultivar CS and barley variety Morex. Further objectives of this paper were: (1) to comprehensively study the effect of barley chromosome arms on agronomic performance and nutrient composition of wheat, and (2) to produce stable and fertile translocation lines that can serve as a genetic basis for improving breeding value of hexaploid wheat.

## Materials and methods

### Plant material

**Fig. 1:**
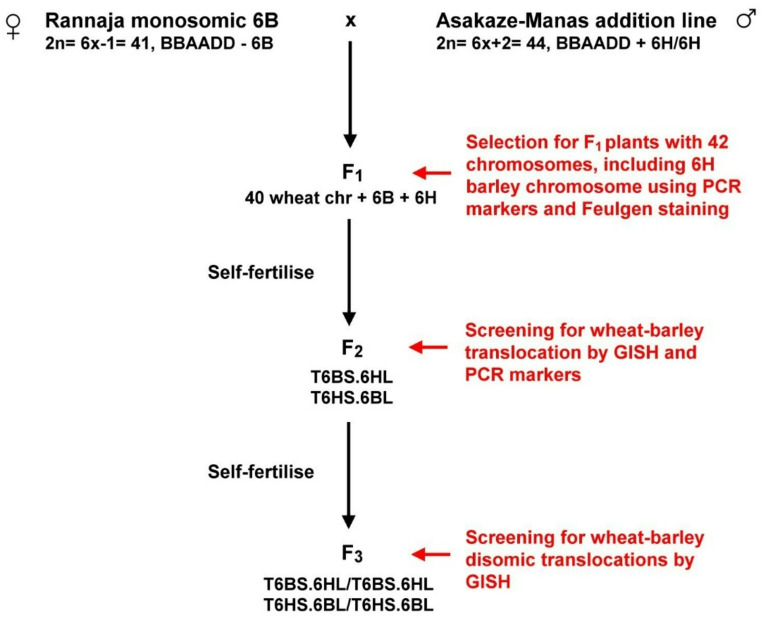
The crossing strategy for development and selection of the T6HS.6BL and T6BS.6HL lines

Two crossing programmes were conducted to introgress barley chromosome segments into the wheat genome, producing stable translocation lines. In the first programme, the winter wheat cultivar Rannaja 6B monosomic stock was crossed as a female parent with the wheat ’Asakaze’-barley ’Manas’ 6H disomic addition line (Fig. 1).

Hybrids with 42 chromosomes, which are double monosomic for chromosomes 6B and 6H were selected in the F_1_ generation using 6H-specific microsatellite markers (*Bmac0316* and *EBmac0806*) and Feulgen staining. After self-pollination of the selected F_1_ plants, the F_2_ progenies were screened using the same microsatellite markers, and then the F_2_ individuals carrying only one arm of chromosome 6H were analysed using GISH to visualize the Robertsonian translocations. The plants containing the translocation in disomic form were also selected with GISH in the F_3_ generation. The wheat (6B) chromosome arm involved in the two translocations was identified using FISH.

**Fig. 2:**
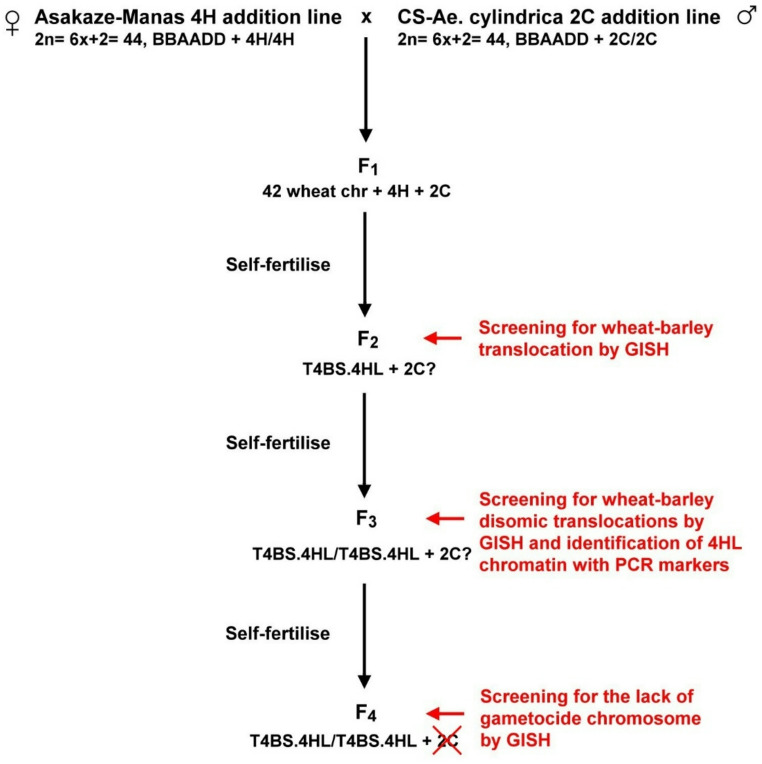
The crossing scheme and development of the T4BS.4HL line

Under the other pre-breeding programme, a cross between Asakaze-Manas 4H and CS-*Ae. cylindrica* 2C addition lines led to the development of F_1_ hybrids, in the descendants of which chromosome 2C is expected to induce structural rearrangement in barley chromosome 4H (Fig. 2).

Genomic DNA of barley was used to carry out GISH on the F_2_ generation genotypes in order to identify plants with the translocation. Individuals of the F_3_ generation containing translocations in homozygous form were also selected using GISH, which were then analysed with FISH and 4H-specific microsatellite markers (*HvM40* and *HvM67*) to identify the wheat and barley parts of the introgression. Genomic DNA from *Ae. cylindrica* was used as a hybridization probe for GISH to check the presence or absence of chromosome 2C in the genetic background of the translocation line.

### Field experiments and agronomic investigation

The field phenotyping trial was conducted at a low-input location (Tükrös nursery, Martonvásár, Hungary; geographic coordinates: 47° 18′ 40″ N, 18° 46′ 56″ E) during the 2023–24 season, which belongs to HUN-REN Centre for Agricultural Research, Agricultural Institute. The T6HS.6BL, T6BS.6HL, and T4BS.4HL lines, along with the Asakaze and Rannaja parental wheat and the Manas barley cultivars, were grown in chernozem soil. In the low-input nursery, which is a pesticide-free location, 50 seeds of each genotype were sown in 5 × 1 m rows (10 seeds per row) with a row distance of 0.15 m. Ten plants per genotype were randomly assigned and selected for characterization of the following morphological traits: plant height (cm), tillering (spikes per plant), seeds per spikelet, length of the main spike (cm), number of seeds per main spike, number of spikelets per main spike and number of seeds per plant. Seed parameters like seed length (cm), width (cm), and thousand-grain weight (TGW) were measured using MARVIN 5.0 Seed Analyzer (MARViTECH GmbH, Germany). Plant height and tillering were recorded in the field immediately before harvest, while the other spike and seed parameters were assessed after harvest. After evaluating the morphological parameters, the seed samples were used for the investigation of grain total amino acid and mineral composition.

### Genomic and fluorescence *in situ* hybridization

Germinating seeds of the wheat-barley translocation lines were grown in a hydroponic system, in which root tip meristem cells were synchronised using hydroxyurea dissolved in Hoagland solution and accumulated in metaphase using amiprofos-methyl (Vrána et al. 2016a, b). The ‘air dry drop’ approach was used to prepare the chromosome spreads (Kato et al. 2004).

To detect and identify the wheat and barley chromosome segments in the translocations, GISH and FISH techniques were used. For GISH, genomic DNA from *Hordeum vulgare* was used as a probe to detect the barley genome in the wheat-barley introgression lines. Barley DNA was labelled with the BioPrime™ DNA Labeling System (ThermoFisher Scientific, Waltham, USA), however, a modified dNTP mix that included digoxigenin-11-dUTP from Roche was utilized, resulting in red signals. Afa-family (Nagaki et al. 1995), pSc119.2 (Contento et al. 2005), and oligo-pTa71 (Tang et al. 2014) FISH probes were used to identify the wheat chromosomes present in the introgression lines. For the Afa-family and pSc119.2 probe labeling, the BioNick™ Labeling System (ThermoFisher Scientific, Waltham, USA) was used, producing a green signal for pSc119.2 probe, and red signal for Afa-family probe because of the modified dNTP mix that included digoxigenin-11-dUTP. Integrated DNA Technologies (Coralville, USA) synthesized the oligonucleotide probe pTa71. Digoxigenin and biotin were added to the synthetic oligonucleotides at their 5′ ends, and when combined in a 6:5 ratio, a yellow signal was detectable. The hybridization mixture during GISH comprised 100 ng of the labeled barley genomic probes, and 3000 ng of bread wheat DNA as a blocker per slide, and dissolved in a 15 µl mixture of 100% formamide, 20 × SSC, 25% dextran-sulfate, and 10% SDS in a ratio of 5:1:2:0.1. Hybridization was carried out overnight at 42 °C. After capturing GISH images under the microscope, the slides were washed three times for 30 min in 2× SSC at 25 °C to remove the signals. The slides were then re-hybridized using FISH. During FISH, 20 ng of oligo-pTa71, 70 ng of pSc119.2 and Afa-family replaced the barley probe and the blocking DNA, while the other solutions were the same as described for GISH.

After post-treatments, digoxigenin and biotin were detected as red and green signals during the detection phase using streptavidin-Alexa Fluor 488 conjugate (Molecular Probes, Waltham, USA) and Anti-Digoxigenin- Rhodamine Fab fragments (Roche) dissolved in TNB (Tris-NaCl blocking buffer). The chromosomes were counterstained with 2 µg/ml DAPI (4’,6-diamidino-2-phenylindole) and mounted in Vectashield antifade solution (Vector Laboratories).

To monitor chromosome 2C in the T4BS.4HL line, the genomic DNA of *Ae. cylindrica* was used for GISH. The protocol followed was the same as described for GISH performed with barley genomic DNA probe.

The slides were examined using a fully automated, high-throughput Zeiss Axio Imager Z2 upright epifluorescence microscope (Carl Zeiss Ltd.). Images were captured using a MetaSystems CoolCube 4 USB laboratory camera; image analysis was performed using Metafer 4 (version 4.3, automated metaphase image acquisition) and ISIS (image processing) software (MetaSystems GmbH).

### Molecular marker analysis

Genomic DNA was extracted from fresh young leaves (plants at the 2-leaf stage) collected from wheat cultivars Asakaze and Rannaja, barley cultivar Manas, and the T6HS.6BL, T6BS.6HL and T4BS.4HL genotypes, using the Quick Gene-Mini80 (FujiFilm, Japan) with a QuickGene DNA tissue kit (FujiFilm, Japan) according to the manufacturer’s instructions (Cseh et al. 2011). The genomic DNA samples of F_1_ and F_2_ hybrids developed from a crossing of Rannaja 6B monosomic line and the Asakaze-Manas 6H addition lines were used for marker-assisted selection of the translocation lines.

PCR amplification of four barley-specific primer pairs (*Bmac0301*-6HS, *EBmac0806–6*HL, *HvM40*- 4HS, and *HvM67*-4HL) was performed on DNA templates extracted from plant samples to detect the presence of chromosome arms 6HS, 6HL, and 4HL. The PCR reactions were carried out in a final volume of 15 µl contained 20 ng of template DNA, 1.5 µl of 10× key reaction buffer (final MgCl_2_ concentration of 1.5 mmol/l), 200 µmol/l of each dNTP, 0.2 µmol/l of forward and reverse primers, and 0.375 U of TEMPase Hot Start DNA Polymerase (VWR International, Belgium). The PCR profiles, annealing temperatures, and oligonucleotide sequences of the primer pairs are available in the GrainGenes database (https://wheat.pw.usda.gov/GG3/) and are included in Supplementary Table 1. The PCR amplicons were separated using a Fragment Analyzer™ Automated CE System equipped with a 96-Capillary Array Cartridge (Advanced Analytical Technologies, USA). The separated fragments were visualized as digital capillary electrophoresis gel images by using PROsize v2.0. software (Advanced Analytical Technologies, USA) to analyse the size and concentration data of all the genotypes examined.

### GBS library development and read coverage analysis

Genomic DNA was extracted from fresh young leaves (plants at 2-leaf stage) of wheat cultivar Asakaze, barley cultivar Manas, and genotypes T6HS.6HL, T6BS.6HL, and T4BS.4HL (individuals from F_5_ generation) using BioSprint DNA kit (Qiagen Inc.) following the manufacturer’s protocol.

The double-digest restriction site-associated DNA (ddRAD) library was constructed following the protocol described by Yang et al. (2016) with minor modifications. Genomic DNA of the plant samples was digested using restriction enzymes MspI and SphI, and the resulting fragments were ligated to sequences of P1 and P2 adapter sequences with barcodes and primers containing indexes according to Poland et al. (2012b). The pooled samples were size-selected for fragment sizes of 350 to 390 bp using a dye-free BluePippin 1.5% gel cassette (BDF1510) (Sage Science, Beverly, MA, USA). All sublibraries were pooled equimolarly and sequenced on a NovaSeq 6000 platform (Illumina) using the SP reagent kit v1.5 in a 2 × 150 bp configuration at the Institute of Experimental Botany, Czech Academy of Sciences, Olomouc, Chech Republic.

Low-pass genome-skimming approach was applied for covarege analysis using the raw reads of Asakaze wheat (No. reads: 6,885,100; Sum. of length: 1082.6 Mb; Average coverage: 0.076×), Manas barley (No. reads: 10,280,976; Sum. of length: 1611.9 Mb; Average coverage: 0.385×) cultivars, and T6HS.6BL (No. reads: 6,582,951; Sum. of length: 1013.5 Mb; Average coverage: 0.072×), T6BS.6HL (No. reads: 8,126,372; Sum. of length: 1258.3 Mb; Average coverage: 0.089×) and T4BS.4HL lines (No. reads: 3,473,695; Sum. of length: 546.8 Mb; Average coverage: 0.039×), as it was described by Adhikari et al. (2022). A mixed genome of an ’in silico wheat × barley hybrid’ was constructed by concatenating the reference pseudomolecules of CS wheat (IWGSC RefSeq v2.1) (Zhu et al. 2021) and Morex barley (Mascher et al. 2017). Quality-filtered read data from translocation and parental lines were aligned to the reference sequences of this combined genome assembly using HISAT2 v.2.1.0 (Kim et al. 2019), to characterise the chromosome constitution of the translocation lines at high resolution. GBS read coverage analysis—including sequence alignment, read counts normalization, and data filtering—was performed using a high-throughput bioinformatics pipeline following the protocol of Adhikari et al. (2022).

### Evaluation of total amino acid and mineral composition

Protein and mineral composition analyses were performed on the grain samples collected from the T6BS.6HL, T6HS.6BL, T4BS.4HL lines, as well as from the parental wheat (Asakaze and Rannaja) and barley (Manas) varieties. Wholemeal samples were obtained from 10 g of seed per genotype using a Retsch Mixer Mill MM 400 ball mill (Retsch, Haan, Germany), and were immediately refrigerated and stored at − 20 °C prior to use. Whole grain flour samples were subsequently analysed for amino acid and mineral composition at the Central Laboratory of Agricultural and Food Products at the University of Debrecen for further study. Measurements were performed in two technical replicates for each genotype.

Protein concentration was determined using the Kjeldahl method (Lynch and Barbano 1999). Following digestion in sulphuric acid and Selenium-containing catalyst (VWR International Ltd., Lutterworth, Leicestershire, UK), ammonium was distilled using a VEPL UDK-149 Distiller (VELP Scientifica, Usmate Velate, Italy) and titrated with an automatic titrator (Velp Titroline 5000). The protein content was calculated from the nitrogen content. The conversion factor was 6.38. Measurements were repeated when the coefficient of variation (CV%) was above 10%.

For amino acid analysis, 0.5 mg of wholemeal flour was hydrolysed with 5 M HCl at 105 °C for 5 h (Memmert UN55, Buechenbach, Germany), then filtered through a regenerated cellulose filter (0.2 μm, Whatman Spartan syringe filter) and diluted to equal protein concentration. Amino acid quantification was carried out using an automatic AAA 500 amino acid analyser (INGOS Ltd., Prague, Czech Republic), based on low-pressure ion-exchange chromatography with post-column derivatisation using ninhydrin. Photometric detection was performed at two wavelengths: 210 nm and 254 nm. A standard amino acid mixture (INGOS Ltd., Prague, Czech Republic) was used as a reference. Recovery exceeded 95%. The amount of amino acids was expressed as a percentage of the total weight of the wholemeal flour.

A 1 g sample of whole grain flour from each genotype was used to determine the concentrations of trace elements (Ca, Mg, Mn, Fe, Cu, and Zn). Plant samples were digested using the pressure-resistant vessels of a microwave digester system (Milestone Ethos Plus, Italy). To all samples, 10 mL of HNO_3_ and 3 mL of 30% (v/v) H_2_O_2_ were added. Digestion was carried out at 120 °C for 90 min, after which the samples were transferred to a 50 ml volumetric flask with distilled water, homogenised, and filtered (MN 640 W paper; Macherey-Nagel, Germany). Inductively Coupled Plasma Optical Emission Spectroscopy (ICP-OES) was applied on an iCAP 7400 spectrophotometer (ThermoFischer Scientific, USA) to measure elemental concentrations. Calibration was performed using a multi-element standard solution prepared from mono-element standards (VWR International Ltd., Leuven, Belgium). Each data point represents the average of three technical replicates. Data were processed using Qtegra ISDS software (version 2.10, Thermo Fisher Scientific, USA). The wavelenghts used for the measurements were: Ca 183.801 nm {483} (Axial); Cu 324.754 nm {104} (Axial); Fe 238.204 nm {141} (Axial); Mg 202.582 nm {118} (Axial); Mn 259.373 nm {130} (Axial); Zn 213.856 nm {458} (Axial).

### Statistical analysis

The agronomic traits of lines T6BS.6HL, T6HS.6BL, and T4BS.4HL were compared pairwise with those of the parental wheat cultivars Asakaze and Rannaja. One-way analysis of variance (ANOVA) was used to assess differences in agronomic traits among genotypes at a significance level of *p* < 0.05. The chemical composition data on macro- and micronutrients were also evaluated using one-way ANOVA with the same significance threshold (*p* < 0.05). For amino acid content, translocation lines were compared pairwise with the wheat parents using a two-sample *t*-test at three significance levels: *p* < 0.05, *p* < 0.01, and *p* < 0.001.

## Results

### Development and identification of the T6BS.6HL and T6HS.6BL lines

**Fig. 3:**
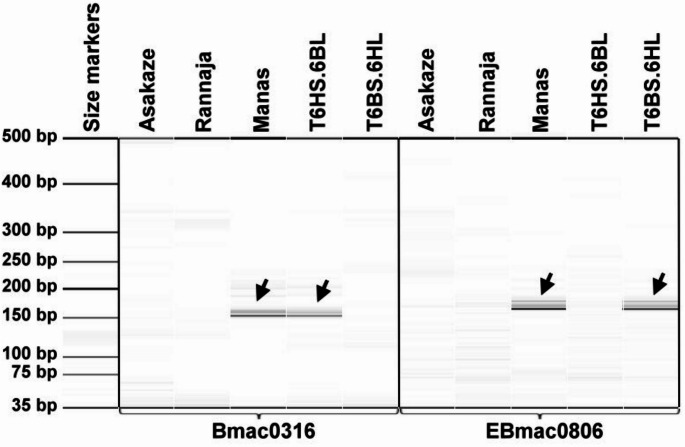
Digital capillary electrophoretic pattern of molecular markers specific to barley chromosome 6H. The *Bmac0316* marker, located on the short arm (6HS), and the *EBMac0806* marker, mapped to the long arm (6HL) of chromosome 6H, were tested on wheat cultivars Asakaze and Rannaja, barley cultivar Manas, and the lines T6HS.6BL/Asakaze/Rannaja and T6BS.6HL/Asakaze/Rannaja. The arrows indicate the presence of barley-specific PCR products in the tested samples. A 35–500 bp DNA ladder was used as a molecular-weight size standard to estimate the fragment sizes

To incorporate barley chromosome 6H into the wheat genome, the Rannaja 6B monosomic wheat line was crossed as a female partner with the Asakaze-Manas 6H disomic addition line. Nineteen F_1_ hybrid plants were screened for the presence of 42 chromosomes, including the barley chromosome, using 6H-specific microsatellite markers (*Bmac0316* and *EBmac0806*) and Feulgen staining. Seven of the nineteen plants carried 42 chromosomes along with the barley 6H chromosome, indicating the double monosomic condition for the chromosomes 6B and 6H (Supplementary Table 2). In the F_2_ generation, forty individuals were screened for the presence of the 6H chromosome arms separately using the same barley-specific microsatellite markers. A total of four plants were produced, only the 6HS-specific PCR fragment, indicating that they carried only the short arm of chromosome 6H. Furthermore, nine plants were found to give a 6HL-specific amplicon, suggesting that they contained only the long arm of chromosome 6H (Fig. 3).

**Fig. 4:**
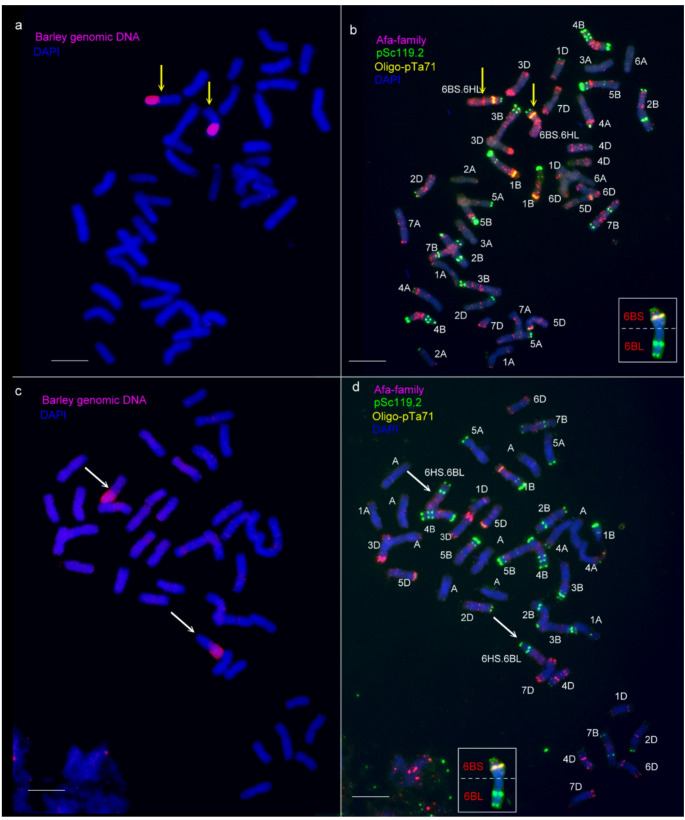
Detection and identification of wheat–barley 6B.6H centric fusion (T6BS.6HL and T6HS.6BL) lines (F3 generation) using genomic in situ hybridization (GISH; **a**,** c**) and fluorescence in situ hybridization (FISH; b, d) on mitotic metaphase chromosome spreads. **a** The barley 6HL arm was detected with barley genomic DNA as a GISH probe, visualized in red. **b** The wheat chromosomes were identified using the Afa-family, pSc119.2, and pTa71 probes during FISH. A T6BS.6HL (yellow arrows) was identified. The inset shows a complete wheat 6B chromosome from a bread wheat karyotype, hybridized with the same FISH probes. This inset allows direct comparison with the T6BS.6HL chromosomes of the introgression lines, clearly indicating the presence of 6BS and the absence of the 6BL chromosome arm. **c** The barley 6HS arm with secondary constrictions was detected with barley genomic DNA as a GISH probe, visualized in red. **d** FISH pattern of the T6HS.6BL line. Inset of 6B allows direct comparison with the T6HS.6BL chromosomes, clearly indicating the presence of 6BL and the absence of the 6BS chromosome arm. DAPI-stained wheat chromosomes appear blue. Wheat–barley centric fusion chromosomes are indicated by arrows. Scale bar = 10 μm

Barley genomic DNA was used for GISH analysis of the F_2_ genotypes selected by molecular markers, identifying three plants (7.5%) carrying a translocation comprising either the long arm (2 plants) or the short arm (1 plant) of chromosome 6H (Supplementary Table 2.). Additionally, telocentric chromosomes were detected in ten other plants, whereas no isochromosomes were observed, indicating that the same chromosome arms of barley and wheat are not fused. Barley chromatin was absent in 21 out of the 40 F_2_ individuals analyzed. To trace the inheritance of the barley chromosome arms incorporated into the wheat genome, 30 plants from each group were also screened by GISH in the F_3_ generation (Fig. 4a and c, Supplementary Fig. S1 and S2). Among these, five genotypes were disomic, 19 were monosomic, and 6 were nullisomic for the translocation involving the short arm of chromosome 6H. Furthermore, eight plants lacked the translocation containing the long arm of chromosome 6H, while three plants carried it in disomic form, and nineteen plants carried it in monosomic form. Repetitive DNA probes were employed in FISH to identify the wheat segments within the translocation lines. In the translocation containing the barley chromosome arm 6HL, the oligo-pTa71 probe—which highlights the secondary constriction at the satellited region of 6BS—produced a typical band on the short arm of chromosome 6B, but produced no signal on the long arm (Fig. 4a). With the help of Afa-family and pSc119.2 repetitive DNA probes, the presence of all other wheat chromosomes were proved (Fig. 4b). Molecular marker and cytogenetic analyses demonstrated that the missing wheat 6BL arm is replaced by the barley 6HL arm, which is fused with 6BS arm to form a Robertsonian translocation, identified as T6BS.6HL (Fig. 4a and b).

In the other translocation containing the barley chromosome arm 6HS, strong terminal and subterminal pSc119.2 signals were detected on the wheat chromosome arm, which is typical for 6BL (Fig. 4d). Therefore, this translocation was identified as T6HS.6BL.

### Identification and selection of the 4BS.4HL translocation line

**Fig. 5:**
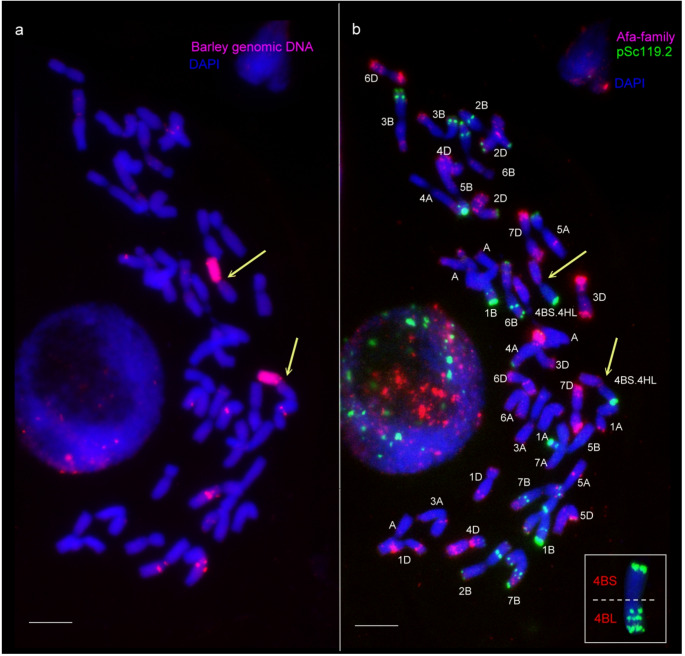
Detection and identification of the T4BS.4HL (centric fusion) line (F3 generation) using genomic in situ hybridization (GISH) and fluorescence in situ hybridization (FISH) on mitotic metaphase chromosome spreads. **a** The entire barley 4HL chromosome arm was detected by GISH and visualized in red. **b** FISH signals on the same cell show a strong pSc119.2 signal, identifying the presence of the wheat 4BS chromosome arm in the centric fusion. DAPI-stained wheat chromosomes are shown in blue. Wheat-barley centric fusions are indicated by yellow arrows. The inset shows a complete wheat 4B chromosome from a bread wheat karyotype, hybridized with the same FISH probes. This inset allows direct comparison with the T4BS.4HL chromosomes of the introgression lines, clearly indicating the presence of 4BS and the absence of the 4BL chromosome arm. Scale bar = 10 μm

To induce rearrangement and develop stable translocation involving barley chromosomes, crosses were made between the Asakaze-Manas 4H and CS-*Ae. cylindrica* 2C addition lines. F_2_ progenies were analysed by GISH using barley genomic DNA as a probe, revealing six genotypes carrying translocations out of 50 plants investigated (Supplementary Table 3.). Additionally, two plants contained a telocentric chromosome, eighteen carried an entire barley chromosome, and one plant possessed both. Barley chromatin was absent in the remaining twenty-three F_2_ plants. Subsequent GISH analysis of 65 F_3_ plants revealed nine disomic and fourteen monosomic individuals for the translocation (Fig. 5, Supplementary Fig. S3 and Supplementary Table 3).

Moreover, eleven plants were detected to carry an intact barley chromosome in addition to either a telocentric or a translocation chromosome. Fourteen F_3_ progeny contained only telocentric or full-length barley chromosomes, while the remaining seventeen plants lacked barley chromatin altogether. A microsatellite marker (*HvM67*), mapped on chromosome arm 4HL, produced a barley-specific PCR fragment in all F_3_ genotypes previously identified as carrying the translocation in either monosomic or disomic form (Supplementary Fig. S4). FISH analysis revealed a prominent telomeric pSc119.2 signal on the translocated wheat chromosome arm, which is typical for 4BS (Fig. 5b inset). No wheat chromosome arm exhibiting the 4BL-specific pSc119.2 pattern (characterised by one telomeric and two interstitial bands, Fig. 5b inset) was detected in these lines (Fig. 5). Based on molecular marker and cytogenetic analyses, this translocation was identified as the T4BS.4HL.

To prevent further chromosomal aberrations and improve the karyotypic stability of the T4BS.4HL line, plants lacking the *Ae. cylindrica Gc* chromosome (2C) were selected. Fifty progeny of F_3_ plants carrying the disomic translocation were subjected to GISH using genomic DNA from *Ae. cylindrica*. The analysis revealed that 25 F_4_ individuals lacked chromosome 2C, while the other half carried it either in homozygous (2 individuals) or heterozygous (23 individuals) form (Supplementary Table 3). In the F_4_ generation, ten progeny derived from a single F_3_ plant were found to lack chromosome 2C. This suggests that the F_3_ parent also did not carry the chromosome with gametocidal effect, and thus its F_4_ progeny may have only minor karyotypic abnormalities. The presence of barley chromatin in these ten F_4_ plants, all from the same lineage, was monitored by GISH, confirming that they carried the translocation in a homozygous form. All three translocation lines along with their parental wheat and barley varieties, were grown and maintained under greenhouse conditions (F_4_–F_6_) and field conditions (F_7_ generation).

### GBS read coverage mapping

GBS platform was used to precisely characterise the extent and structure of the barley chromatin added to the wheat genome. Furthermore, we aimed to thoroughly examine karyotypic changes and instability within the wheat genetic background, which commonly accompany chromosome manipulation methods, particularly those involving the gametocidal system. Illumina short-reads generated from the wheat (Asakaze) and barley (Manas) control genotypes, as well as from the three translocation lines (T6HS.6BL, T6BS.6HL, and T4BS.4HL) were aligned to the reference sequences of the combined wheat-barley genome. When sequence tags from the wheat parental variety (Asakaze) were mapped onto this mixed reference, high normalized read values (0.865–1.197 per Mb bin) were detected across the twenty-one wheat chromosomes of the in silico hybrid, while low read densities (0.009–0.013 per Mb bin) were observed on the seven barley chromosomes (Supplementary Fig. S5 and Supplementary Table 4.). This indicates that the wheat control genotype (Asakaze) showed strong coverage across the CS wheat reference pseudomolecules, making it suitable for mapping wheat chromosomes of the translocation lines. Similarly, the short-read sequences from the barley parent (Manas) exhibited high normalized read values (1.338–1.416 per Mb bin) along the barley chromosomes, while low read values (0.002–0.006 per Mb bin) were observed on the wheat chromosomes (Supplementary Fig. S6 and Supplementary Table 4.). Given the high coverage of barley reads across the Morex barley reference pseudomolecule, this combined in silico hybrid genome is appropriate for accurately determining the extent of barley chromatin introgression.

The short-read sequences of all three translocation lines were detected in dense coverage (normalized read values ranged from 0.516–1.634 per Mb bin) on almost all chromosomes of the ABD genome of the in silico hybrid, except the 4BL or 6B chromosome arms.

### T6HS.6BL line

**Fig. 6:**
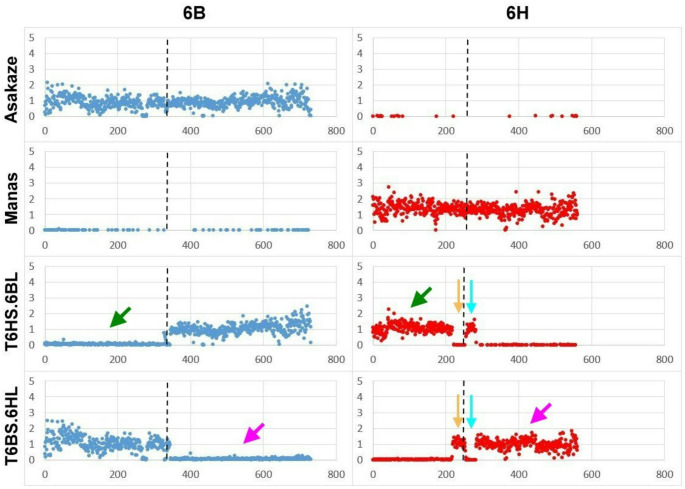
Normalized GBS read coverage of the wheat (Asakaze) and barley (Manas) parental cultivars, and the T6HS.6BL and T6BS.6HL lines (F_5_ generation) along the 6B and 6H chromosomes of the in silico hybrid. The x-axis represents the genomic position within the chromosome in Mb, while the y-axis shows the normalized read coverage. Green arrows mark the incorporation of the 6HS chromosome arm in the place of 6BS arm in line T6HS.6BL, while purple arrows indicate that the 6HL chromosome arm replaces the 6BL arm in line T6BS.6HL. Orange arrows indicate a small, 36 Mb-long region (between 218–254 Mb) that is missing from the T6HS.6BL line but is present in the T6BS.6HL line. Turquoise arrows mark the adjacent 28 Mb-long region (between 255–283 Mb) that is present in the T6HS.6BL line but is missing from the T6BS.6HL line. Centromere positions are marked by a black dashed line

In terms of the T6HS.6BL line, an extremely low normalized read value (0.06 per 1 Mb) was observed on the first 345 Mb of the 6B in silico chromosome (Fig. 6, Supplementary Fig. S7 and Supplementary Table 4.). This wheat chromosomal interval is equal to the length of 6BS arm, which is missing from the translocation (Šafář et al. 2010; Zhu et al. 2021).

**Fig. 7:**
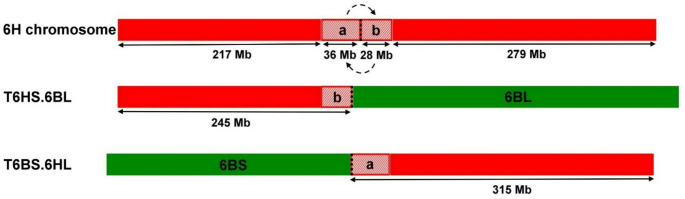
Comparison of the introgressed chromosomes in T6HS.6BL and T6BS.6HL lines with barley chromosome 6H based on short-read mapping to the combined genomes of wheat (CS) and barley (Morex). Barley and wheat segments are shown in red and green, respectively. A 36 Mb (**a**) and a 28 Mb (**b**) barley-derived segment were identified flanking the main 6HS (217 Mb) and 6HL (279 Mb) regions, respectively. In T6BS.6HL, the barley chromatin includes regions (**a**) and 6HL, but lacks segment (**b**). In contrast, T6HS.6BL carries regions (**b**) and 6HS, but lacks segment (**a**). The two small segments (**a** and **b**) are reversed in position relative to their original location on chromosome 6H. Centromere positions are marked by a black dotted line

A high normalized read coverage (1.079 per 1 Mb) was revealed along the first region (217 Mb) of the 6H in silico chromosome (Fig. 6 and Supplementary Fig. S7 and Supplementary Table 4.). However, a low average rate (0.009 per 1 Mb) was observed across the following 36 Mb region, which was followed by a 28 Mb interval exhibiting a high normalized read value (1.021 per 1 Mb). A low density of reads (0.011 per 1 Mb) was detected along the last 279 Mb long region, indicating that the latter segment is absent from the translocation, which covers most of the 6HL chromosome arm (Suchánková et al. 2006; Mascher et al. 2017). The read coverage analysis of the T6HS.6BL line showed that the introgressed 6H chromatin consists of a 217 Mb and a 28 Mb long segment, and these two fragments delimit a section of 36 Mb length that is missing from the translocation (Figs. 6 and 7, Supplementary Table 4). The length of barley chromatin involved in the T6HS.6BL line is 245 Mb-including the 217 and 28 Mb regions-, which comprises about 44% of the whole chromosome (560 Mb). This is largely consistent with the short arm, which accounts for 48% of the total chromosome size (Suchánková et al. 2006). The finding obtained from GBS read coverage mapping confirmed the results of the molecular marker and cytogenetic analyses: the missing wheat 6BS arm is replaced by a large part of the barley 6HS arm, forming the 6HS.6BL translocation together with the entire wheat 6BL arm.

### T6BS.6HL line

In the case of the T6BS.6HL line, a high normalized read value (1.032 per Mb) was detected along the first 345 Mb region of chromosome 6B of the combined genome. In contrast, only an average value of 0.055 was found along the last 386 Mb region (Fig. 6, Supplementary Fig. S8 and Supplementary Table 4.), suggesting that the wheat fragment covering the length of the short arm is involved in the translocation, whereas the wheat segment corresponding to the long arm is missing (Šafář et al. 2010; Zhu et al. 2021). A very low normalized read value (0.014 per 1 Mb) was revealed along the first 217 Mb interval of the 6H in silico chromosome. At the same time, a high average rate (1.070 per 1 Mb) was observed in the subsequent 36 Mb region (Fig. 6, Supplementary Fig. S8 and Supplementary Table 4.). A low density of short-reads (0.007 per 1 Mb) was found in the next 28 Mb long region, whereas high normalized read coverage (0.980 per 1 Mb) was detected along the final 279 Mb region. As a result, the 36 Mb and 279 Mb segments with high read coverage constitute the barley chromatin in the T6BS.6HL line (Figs. 6 and 7, Supplementary Table 4.), which accounts for approximately 56% of the entire chromosome (561 Mb). This corresponds mainly to the long arm, which comprises 52% of the total chromosome length (Suchánková et al. 2006). GBS read coverage mapping demonstrated that the entire 6BS arm of wheat and most of the the 6HL arm of barley–replacing the 6BL arm–constitute the 6BS.6HL translocation, supporting the findings of previous analyses.

### T4BS.4HL line

**Fig. 8:**
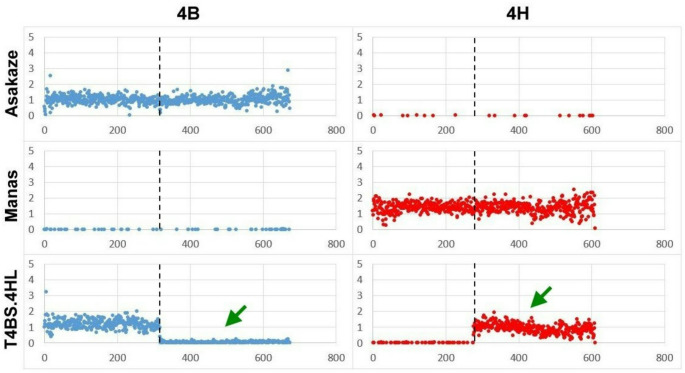
Normalized GBS read coverage of the wheat (Asakaze) and barley (Manas) parental cultivars, and the T4BS.4HL line (F_5_ generation) along the 4B and 4H chromosomes of the in silico hybrid. The x-axis represents the genomic position within the chromosome in Mb, while the y-axis shows the normalized read coverage. Green arrows indicate the presence of the 4HL chromosome arm in the T4BS.4HL line, while the 4BL arm is absent. Centromere positions are marked by a black dashed line

For the T4BS.4HL line, a small number of reads (normalized read value of 0.054 per Mb bin) were mapped onto the last 356 Mb region of the in silico 4B chromosome (Fig. 8, Supplementary Fig. S9 and Supplementary Table 4.), indicating that the entire 4BL arm is missing from the translocation (Šafář et al. 2010; Zhu et al. 2021). In contrast, a larger normalized read coverage (0.927 per Mb bin) was detected along the last 335 Mb of the 4H in silico chromosome, proving that the barley fragment covering the entire 4HL arm is involved in the translocation (Fig. 8 and Supplementary Table 4.). This is also consistent with the results of SSR markers and cytogenetic analyses, confirming the presence of the 4BS.4HL translocation.

In the T4BS.4HL line, low normalized read values (ranged from 0.010–0.233 per Mb bin) were detected along a 49 Mb long region (573–622 Mb) of chromosome 2D and the last 127 Mb region (724–851 Mb) of chromosome 3B, indicating the presence of large deletions in the wheat genetic background (Supplementary Fig. S9). A large number of GBS reads provided high coverage and density for both the parental and translocation lines, which were mapped onto the combined reference genome of the in silico hybrid, ensuring detailed characterization of the introgressed barley chromosomes and the wheat genetic background.

### Morphological characterization

To assess whether the barley chromosome arms compensate for the missing wheat chromosome arms in the newly identified homoeologous centric fusions, we compared morphological parameters between parental wheat and translocation lines.

The plants were grown in a low-input field (Tükrös Nursery), under weather typical of a continental climate. During the 2023–24 growing season, the experimental area received 460 mm of precipitation, including 120 mm of rainfall during the grain-filling period. Plant height of the T6BS.6HL and T6HS.6BL lines was considerably higher than that of the wheat control varieties and the T4BS.4HL line (Table 1). Furthermore, the T6HS.6BL line exhibited the longest main spike, containing a slightly higher number of seeds compared to the wheat parents and the other two translocation genotypes. Tillering (number of spikes per plant) was highest (but not significantly) in the T6BS.6HL genotype, while the other two translocation lines displayed values similar to those of the ‘Asakaze’ and ‘Rannaja’ wheat controls. A higher number of spikelets per main spike was observed in all three translocation lines compared to the Asakaze and Rannaja wheat varieties. However, no significant differences were found between the translocation lines carrying 6H chromosome arms and the wheat parental cultivars in the number of seeds per main spike or fertility (number of seeds per spikelets on the main spike), except for the T4BS.4HL line, which showed the lowest values for these traits. The number of seeds per plant for all three translocation lines was similar to that of the ‘Asakaze’ wheat parent, whereas the ‘Rannaja’ wheat control exhibited significantly lower yield.

**Table 1:** Morphological traits of the Asakaze and Rannaja parental wheat cultivars and the three translocation lines

	Asakaze	Rannaja	T6HS.6BL	T6BS.6HL	T4BS.4HL
Plant height (cm)	93.6 ± 4.6 ^c^	86.1 ± 4.2 ^d^	**107.4 ± 4.3** ^a^	**99.1 ± 4.1** ^b^	90.2 ± 10.1 ^cd^
No. spikes per plant	4.8 ± 0.8 ^ab^	3.4 ± 0.8 ^c^	4.4 ± 1.2 ^bc^	5.5 ± 0.8 ^a^	4.7 ± 0.7 ^b^
Length of main spike (cm)	8.4 ± 1.1 ^b^	8.1 ± 0.6 ^b^	**10.1 ± 0.6** ^a^	8.3 ± 1.5 ^b^	8.1 ± 0.9 ^b^
No. spikelets per main spike	18.2 ± 0.7 ^b^	19.1 ± 1.2 ^b^	**20.8 ± 1.4** ^a^	20.7 ± 2.6 ^ab^	**22.2 ± 2.1** ^a^
No. seeds per main spike	41.8 ± 6.9 ^ab^	43.8 ± 7.8 ^ab^	49.3 ± 5.2 ^a^	45.1 ± 13.3 ^ab^	37.2 ± 9.0 ^b^
No. seeds per spikelets	2.3 ± 0.3 ^a^	2.3 ± 0.3 ^a^	2.4 ± 0.2 ^a^	2.4 ± 0.4 ^a^	1.7 ± 0.4 ^b^
No. seeds per plant	154 ± 38 ^ab^	126 ± 18 ^b^	161 ± 45 ^ab^	167 ± 44 ^a^	166 ± 26 ^a^
Grain length (mm)	6.6 ± 0.2 ^ab^	6.6 ± 0.3 ^ab^	6.8 ± 0.2 ^a^	6.4 ± 0.2 ^b^	6.4 ± 0.2 ^b^
Grain width (mm)	3.4 ± 0.1 ^b^	3.7 ± 0.1 ^a^	3.5 ± 0.2 ^b^	3.3 ± 0.1 ^b^	3.7 ± 0.1 ^a^
Thousand-grain weight (g)	37.5 ± 4.4 ^b^	41.5 ± 3.2 ^ab^	42.8 ± 3.9 ^a^	37.0 ± 4.1 ^b^	41.0 ± 4.1 ^a^

Data expressed as mean ± SD of 10 plants per genotype for each agronomic parameter. Different letters indicate significant differences between the genotypes at *p* < 0.05, as determined by one-way ANOVA. The boldface values of the translocation lines indicate significantly better agronomic performance compared to the wheat parents

None of the translocation lines exhibited seed-related traits (such as grain width and length) that were significantly higher than those of the wheat varieties; consequently, their thousand-grain weight (TGW) values were also similar. The T6HS.6BL/Asakaze/Rannaja translocation line developed long-awned spikes resembling those of the Asakaze wheat cultivar. In contrast, the T6BS.6HL/Asakaze/Rannaja line produced shorter, awned main spikes, similar to the Rannaja cultivar. The spikes of the T4BS.4HL/Asakaze translocation genotype, characterised by short awn stubs, showed a high degree of similarity to those of the Asakaze wheat parental cultivar (Supplementary Fig. S10).

### Analysis of the amino acid and mineral composition

We were interested not only in whether the incorporated barley chromosome arms functionally replace their wheat counterparts, but also whether they could enhance the quality traits of bread wheat. To investigate the effect of introgressed barley chromosome arms on the amino acid composition of wheat grain, the wheat-barley translocation lines were compared with wheat varieties Asakaze and Rannaja. The T4BS.4HL genotype contained significantly higher levels of all essential amino acids, except lysine, compared to both the wheat cultivars and the other two translocation genotypes (T6HS.6BL and T6BS.6HL). Additionally, the Manas barley parent exhibited the lowest proportion of these protein compounds among all tested genotypes, suggesting that the increased amino acid content in the T4BS.4HL line may be not solely due to the presence of barley chromatin, but rather to the combined effect of wheat and barley alleles (Table 2).

**Table 2:** Essential amino acid content (m/m%) in wholemeal samples of wheat cultivars Asakaze and Rannaja, barley cultivar manas and the three translocation lines

	Asakaze	Rannaja	Manas	T6HS.6BL	T6BS.6HL	T4BS.4HL
THR	0.36 ± 0.01	0.29 ± 0.03	0.33 ± 0.01	0.29 ± 0.01	0.35 ± 0.01	0.44 ± 0.01***
VAL	0.54 ± 0.03	0.47 ± 0.03	0.45 ± 0.03	0.46 ± 0.00	0.55 ± 0.05	0.67 ± 0.04*
MET	0.11 ± 0.01	0.06 ± 0.01	0.07 ± 0.00	0.05 ± 0.00	0.10 ± 0.01	0.18 ± 0.01***
ILE	0.41 ± 0.04	0.36 ± 0.00	0.29 ± 0.01	0.39 ± 0.01	0.41 ± 0.01	0.54 ± 0.02**
LEU	0.83 ± 0.06	0.76 ± 0.05	0.59 ± 0.02	0.93 ± 0.03	0.90 ± 0.08	1.10 ± 0.02**
PHE	0.53 ± 0.04	0.46 ± 0.04	0.40 ± 0.01	0.45 ± 0.04	0.45 ± 0.04	0.78 ± 0.07**
HIS	0.28 ± 0.01	0.24 ± 0.02	0.19 ± 0.02	0.32 ± 0.03	0.31 ± 0.01*	0.38 ± 0.02***
LYS	0.48 ± 0.04	0.41 ± 0.01	0.45 ± 0.03	0.53 ± 0.01	0.51 ± 0.03	0.53 ± 0.01

THR, threonine; VAL, valine; MET, methionine; ILE, isoleucine; LEU, leucine; PHE, phenylalanine; HIS, histidine; LYS, lysine. Data are expressed as the mean ± SD per genotype for each parameter

*, **, *** indicate significant difference from the corresponding wheat parent at *p* < 0.05, *p* < 0.01 and *p* < 0.001 levels, respectively, using two-sample *t*-test

Differences in mineral content were also analysed between the translocation lines and their parental wheat and barley cultivars. The T6BS.6HL line exhibited a significantly higher calcium (Ca) content compared to both the wheat and barley parents, as well as the other two translocation lines. Furthermore, the Ca concentration in the Manas barley was considerably higher than in the wheat cultivars Asakaze and Rannaja, suggesting a possible positive effect of the barley 6HL chromatin (Table 3).

**Table 3:** Mineral content (mg/kg) in wholemeal samples of wheat cultivars Asakaze and Rannaja, barley cultivar Manas and the three translocation lines

	Asakaze	Rannaja	Manas	T6HS.6BL	T6BS.6HL	T4BS.4HL
Ca	573 ± 1 ^cd^	576 ± 3 ^cd^	643 ± 1 ^b^	580 ± 1 ^c^	**775 ± 0.2** ^a^	568 ± 2 ^d^
Cu	5.77 ± 0.10 ^b^	6.44 ± 0.13 ^a^	5.24 ± 0.11 ^bc^	6.30 ± 0.02 ^a^	5.06 ± 0.06 ^c^	4.71 ± 0.01 ^d^
Fe	39.32 ± 0.40 ^d^	39.07 ± 0.50 ^d^	43.99 ± 0.12 ^c^	**58.34 ± 0.27** ^a^	44.46 ± 0.36 ^c^	**56.23 ± 0.34** ^b^
Mg	1439 ± 18 ^b^	1542 ± 41 ^ab^	1358 ± 31 ^b^	**1681 ± 22** ^a^	**1710 ± 17** ^a^	1337 ± 17 ^bc^
Mn	48.08 ± 0.16 ^b^	41.31 ± 0.33 ^d^	17.77 ± 0.15 ^e^	**55.92 ± 0.10** ^a^	46.29 ± 0.29 ^c^	48.39 ± 0.05 ^b^
Zn	41.06 ± 0.06 ^c^	37.99 ± 0.12 ^d^	30.57 ± 0.57 ^e^	**44.32 ± 0.23** ^a^	31.49 ± 0.38 ^e^	42.05 ± 0.08 ^b^

Data are expressed as mean ± SD per genotype for each parameter. Different letters indicate significant differences between the genotypes at *p* < 0.05, as determined by one-way ANOVA. The boldface values of the translocation lines indicate significantly higher trace element content compared to the wheat parents

The T6HS.6BL and T4BS.4HL lines contained significantly higher amounts of iron, approximately 48 and 12% more, respectively, compared to the wheat and barley parents. Among the parents, the Manas barley had higher iron levels than the Asakaze and Rannaja wheat cultivars. This finding supports that the increase in iron content could be resulted from the introgression of the barley 6HS and 4HL chromosome arms. The T6HS.6BL line also contained notably higher proportions (ranging from 8–16%) of Mg, Mn and Zn compared to its wheat and barley parental cultivars. In contrast, the T6BS.6HL genotype, along with its barley parental line, showed much lower zinc levels than wheat lines, which could be attributed either to the introgression of barley 6HL chromatin or to the loss of the wheat 6BL chromosome arm.

## Discussion

The majority of the population in low-income countries consumes predominantly staple crops such as rice and bread wheat that are extremely poor in trace elements. Moreover, peoples from these indigent social classes rarely have access to foods that are ordinary fortified with added nutrients. Biofortification may be a future solution for dealing with nutrient deficiencies in developing countries, where anemia caused by a lack of iron is one of the most common health problem (Garg et al. 2018). The nutritional values of cereals can be increased through conventional selective breeding, or through genetic modification, but the latter is quite contraversial for society. Introgression breeding as conventional technique is a widely accepted strategy for transferring agronomically useful gene variants from related species into bread wheat. Barley can be used as a promising hybridization partner, whose 4H and 6H chromosomes provide a suitable genetic basis for increasing the trace element content (especially iron) of bread wheat. Although morphological parameters and nutritional values were examined in this work, it is worth noting that these barley chromosomes have a major influence also on drougth tolerance-related traits, which is supported by the results found in several studies (Handley et al. 1994; Teulat et al. 1998, 2001, 2003; Molnár et al. 2007). In the present study, we developed and identified stable wheat-barley Robertsonian translocation lines carrying the 4H and –6H chromosome arms, which could potentially contribute to improving the nutritional composition of bread wheat and may also facilitate climate-resilient breeding.

So far, only a few compensating wheat-barley translocation lines have been published that can be used for wheat breeding. Using model wheat and barley genotypes, Molnár-Láng and Sutka (1994) developed a 3HS.3BL centric fusion by hybridization of ‘CS’ wheat and ‘Betzes’ spring barley cultivars, which was later shown to improve tillering and seed productivity in hexaploid wheat (Türkösi et al. 2015). In addition, Danilova et al. (2018) produced a complete set of homoeologous group 7 compensating translocations, transferring chromosome arms from ’Betzes’ barley into the ’CS’ wheat genome. Using wheat and barley cultivars with more advanced agronomic values, Türkösi et al. (2018) developed a T7BS.7HL centric fusion through the double monosomic strategy to induce centric breakage-fusion between the chromosomes of the elite cultivars ‘Manas’ barley and ‘Rannaja’ wheat. This translocation exhibited and elevated grain β-glucan content compared to the parental wheat cultivars. Using a similar approach for the production of Rannaja-Manas homoeologous centric fusions, the present work supports that the breakage-fusion mechanism of univalent chromosomes is a feasible approach to transfer novel agronomically advantageous gene variants into wheat.

Taking advantage of the centric breakage and fusion mechanism, we found a similar proportion (7.5%) of plants carrying Robertsonian translocations as reported by Türkösi et al. (2018). In contrast, Danilova et al. (2018) observed a much lower efficiency (0.5–0.9%), which can also be explained by the fact that different wheat (CS or Rannaja, Asakaze) and barley (Betzes or Manas) varieties were used as crossing partners for intergeneric hybridization. Utilizing effect of the gametocidal system, tranlocation were observed in 12% of the F_2_ plants examined, which is consistent with the results reported by Shi and Endo (1999), who detected introgressions containing the 4H barley chromosome at a similar frequency (17.6%).

Chromosome breaks have been induced to produce translocations; however, the chromosome manipulation strategies can lead to further structural changes in the genetic background. GBS read coverage analysis was used to check these karyotypic abnormalities, with a particular focus on aberrations caused by the 2C gametocidal chromosome. The GBS mapping not only confirmed the findings obtained from cytogenetic and molecular marker analyses by identifying the wheat and barley chromosome arms in the centric fusions, but also verified that the wheat chromosome set in the T6HS.6BL and T6BS.6HL lines is undamaged and does not contain deletion of major regions (Supplementary Fig. S7 and S8). In contrast, two larger deletions were detected in the genetic background of the T4BS.4HL line (Supplementary Fig. S9), which are located on wheat chromosomes 2D (573–622 Mb) and 3B (724–851 Mb).

Coverage analysis revealed a 64-Mb region (217–281 Mb) along the centromere of chromosome 6H, two segments of which (28 Mb and 36 Mb) have changed places in the barley chromosome arms of the translocation lines relative to those of the barley reference genome (Fig. 7). One possible explanation for this is that the Morex barley reference pseudomolecule may contain sequence scaffolds near the centromere that have not yet been assembled in the correct order. Because of the lower probability of meiotic recombination close to the centromere, genetic mapping and thus precise estimation of the distance and order of markers is rather difficult in this region (Künzel et al. 2000). Since the two chromosome segments are located in the pericentromeric–centromeric region, it is possible that their short-reads were aligned to the proximal end of the opposite chromosome arm. However, the centromere of barley chromosomes was characterised in detail when the sequence fraction of individual chromosome arms flow-sorted from the wheat-barley telosomic addition lines (2HS-7HL) was used to resolve the linear gene order (Mayer et al. 2011). As a result, all but nine of the 3125 centromere-specific genes were assigned to either the short or long arm of a barley chromosome (Mayer et al. 2011). Moreover, new approaches such as optical mapping and chromosome conformation capture sequencing (Hi-C) provide high-quality assembly for the barley reference genome and enable accurate chromosomal localization of sequence scaffolds even in the pericentromeric region (Mascher et al. 2017). Thus, it is more likely that the chromosomal position of the two segments was exchanged due to a pericentric inversion that occurred before the centric breakage and fusion of the 6H chromosome. Consequently, the putative inversion spanned the centromere, resulting in a breakpoint in each chromosome arm. It remains a question whether this possible pericentric inversion is inherently present in the genome of Manas barley variety or is only found in that of the two complementary translocations. The GBS read coverage analysis of chromosome arms 6HS and 6HL in the Asakaze-Manas ditelosomic addition lines (Türkösi et al. 2016) may provide further insight into chromosome rearrangements in the pericentric region.

Chromosomal inversions have a significant impact on wheat evolution and breeding, primarily by drastically altering the frequency of meiotic recombination (Stevison et al. 2011; Termolino et al. 2019). These structural variations in heterozygous form effectively suppress recombination events within the inverted chromosomal region (Wellenreuther and Bernatchez 2018; Todesco et al. 2020). Low recombination rates lead to the formation of haplotype blocks that contain gene clasters consisting of advantageous and disadvantageous alleles at multiple linked loci (Thompson and Jiggins 2014). On the one hand, inversion can carry a favorable combination of alleles that regulates complex traits such as disease resistance, drought tolerance, or yield components, which is inherited as one unit, making it extremely beneficial for local adaptation and breeding strategies (Kirkpatrick 2010; Cao et al. 2024). The low recombination rate may facilitate the stabilization and preservation of wild adaptive gene complexes within inversions after they have successfully introgressed into the wheat genome (Hoffmann and Rieseberg 2008).

However, these non-recombination blocks can also carry potentially harmful alleles (negative traits) linked to beneficial alleles (Connallon and Olito 2021). The linkage drag makes it difficult for breeders to separate these coupled gene variants, particularly within pericentromeric inversions, where low recombination rate poses a significant challenge for the accurate identification of quantitative trait loci (QTLs). Understanding the effects of chromosomal inversions is extremely important for the introgression breeding of hexaploid wheat. In wheat breeding, these structural changes are an obstacle due to the linkage drag, but they also offer an opportunity to exploit the positive, co-adapted gene complexes for crop improvement.

To detect structural changes in the smaller chromosomal regions, a sufficient number of uniplex markers must be tested to ensure dense coverage, which is a rather expensive requirement. The GBS technology is becoming increasingly a cost-effective platform that provides detailed sequence information for the identification of minor chromosomal rearrangements, such as inversions, deletions or insertions. Some publications have already reported that the GBS platform was utilized successfully for the detection of chromatin segments in the wheat genome, which were introgressed from cultivated and wild relatives, including *Aegilops biuncialis* (Gaál et al. 2024), *Aegilops umbellulata* (Bansal et al. 2020), perennial rye (Szakács et al. 2020) and *Agropyron glael* (Kruppa et al. 2025), which is a hybrid of *Thinopyrum intermedium* and *Th. ponticum*. Castillo et al. (2013) performed Diversity Array Technology (DArT), a modified GBS method for identifying the substitutions of chromosomes D/H^ch^ in wheat-*Hordeum chilense* amphiploids. Adhikari et al. (2022) applied a high-throughput genotyping platform (skim-sequencing) to characterise group-7 translocation chromosomes in wheat-barley recombinant lines. In the present work, we have also demonstrated that it can serve as a powerful approach, even replacing the use of molecular markers and cytogenetic methods, to accurately map introgression lines produced by interspecific hybridization.

Investigation of agronomic parameters showed that the presence of short and long arms of chromosome 6H may have a positive correlation with tallness, which is confirmed by the results of several publications that have reported a strong association between the QTLs on this barley chromosome and plant height. Pu et al. (2021) mapped a larger region (*HvSS1*) on both arms of chromosome 6H (173–396 Mb), which regulates uppermost internode elongation and thus determines plant height in barley. Pasam et al. (2012) analysed the relationship between the genomic regions and agronomic traits within a collection of 224 spring barley accessions. They identified three putative QTLs on the 6H chromosome (12.5 cM, 55.3 cM and 124.8 cM) that influencing plant height. Hu et al. (2018) detected a SNP (*qtncIL3-6H-1*) on the short arm of chromosome 6H (16.1–17.5 bp) that is strongly associated with a plant height component trait (internode length). The short arm of barley chromosome 6H could have a positive effect not only on plant height but also on spike length, which is in agreement with previous studies. Gyenis et al. (2007) identified a QTL on the chromosome arm 6HS (BINs 5 and 6), that increased spike length. Jabbari et al. (2018) found a putative QTL (*D2Q1MSL6H*) on chromosome 6H, controlling the significant spike length of spring barley cultivars under drought stress.

There were no significant differences between the T6BS.6HL and T6HS.6BL lines and wheat parental varieties in most morphological and yield-related traits, which is consistent with the results of GBS analysis that the chromosomal set of their wheat genetic background did not contain major deletions. In contrast, the much lower fertility of the T4BS.4HL genotype is likely due to the gametocidal effect, which may be explained by the presence of two larger deletions on the 2D and 3B wheat chromosomes. Nevertheless, it should be emphasized that the morphological characterisation relies on data from a single season and location, therefore long-term field trials will be required to validate the stability and agronomic benefit of the translocation lines.

Development of stable translocation lines also provides an opportunity to study the effect of barley chromosome arms on the quality traits of bread wheat. Of the three translocations, only the T4BS.4HL line showed a significant increase in the level of essential amino acids (except lysine). Since the amino acid content of the barley control Manas was lower than that of the wheat parents, it can be concluded that the 4HL chromatin may not act alone but rather in combination with the wheat alleles, may be responsible for the change in protein composition in the translocation line. Information about the genomic regions on barley chromosome 4H that affect amino acid levels has not yet been reported. Oddy et al. (2023) identified QTLs on the proximal (327 Mb) and distal (601, 547 and 518 Mb) parts of wheat chromosome arm 4BL, which are responsible for controlling glycine, glutamine, glutamic acid and asparagine. In the T4BS.4HL line, the barley chromosome arm may likely not only replace these wheat candidate genes but also contribute through novel allelic combinations to alterations in protein composition. However, since field experiment is limited to a single year, multi-environment trials are needed to draw back long-term conclusion about the possible effect of wheat-barley introgressions on the amino acid composition.

A wide variability in the concentration of multiple elements has been observed within different barley populations (Mamo et al. 2014; Gyawali et al. 2017; Nyiraguhirwa et al. 2022), suggesting that the integration of barley chromatin into the wheat genome may induce changes in nutrient composition. In the T6BS.6HL line, we found that the barley chromosome arm has a possible positive effect on Ca content. There is no direct evidence that the 6H chromosome influences Ca levels; however, two genes (*rhi1* and *rhs2*) were mapped earlier in the centromere region, regulating the phenotypes of barley root hairs, and thereby macronutrient uptake (Janiak and Szarejko 2007; Chmielewska et al. 2014). The present work has shown that introgression of chromosome arms 6HS and 4HL may have result in significant increases in iron and zinc concentrations, which is in agreement with the findings of some publications that reported a correlation between these genomic regions and micronutrient levels in barley (Uauy et al. 2006; Distelfeld et al. 2008; Nyiraguhirwa et al. 2022). The *TtNAM-B1* gene plays a role in several functions, one of which is to accelerate the transport of microelements from leaves into the grains (Uauy et al. 2006). Its orthologous variants are located on 6BS and 6HS chromosome arms, thus, replacing the wheat allele with that of barley (*HvNAM-1*) could result in elevated iron and zinc levels in the T6HS.6BL line (Uauy et al. 2006; Distelfeld et al. 2008). Nyiraguhirwa et al. (2022) identified two genomic regions in the long arm of barley chromosome 4H (615 and 598 Mb), one of which was associated with zinc, and the other with iron content, suggesting that the barley chromatin may be responsible for the change in the microelement levels in the T4BS.4HL line. Velu et al. (2017) detected a QTL (*Qzneff.sar_6B*) on the long arm of wheat chromosome 6B, conferring high zinc concentration. In addition, Mamo et al. (2014) identified two QTLs on chromosome 6HL (122.9 and 128.7 cM) that affect zinc content in barley grain. The low level of zinc in the T6BS.6HL and barley control genotypes could suggest that barley alleles are not able to replace the loss of the wheat 6BL chromosome arm functionally. Although the functional genes regulating the metabolism of minerals and amino acids were not under examination in the present work, the translocation lines provide helpful information for understanding the expression of allelic variants from barley in the wheat genetic background. Nonetheless, it must be emphasized that the agronomic evaluation is restricted to a single year and location, therefore, additional environmental trials is required to confirm the agronomic advantage of the newly developed lines. Field experiments conducted under multiple environmental conditions and with several replications would allow a more reliable investigation of the effect of the translocations on trace-element composition.

## Conclusions

The incorporation of barley chromosome arms 4H and 6H into the wheat genome has led to the development of Robertsonian translocations with stable inheritance. Their similar agronomic performance compared to the parental wheat varieties indicates that introgressions have a negligible deleterious effect in the wheat genetic background, and their wheat chromosome set does not contain major deletions, except for the 4BS.4HL centric fusion line, which lacks two larger regions (49 and 127 Mb) from chromosomes 2D and 3B. In the present work, we have found that introgressed barley chromosome arms not only compensate for the lack of wheat chromosome arms but also improve nutrient composition (especially Fe content) in the newly developed translocation lines. In situ hybridizations with barley genomic DNA probe and oligonucleotide DNA repeats, as well as PCR analysis with barley-specific molecular markers, were used to identify the wheat and barley chromosome arms in the compensating translocations, which were designated as T4BS.4HL, T6HS.6BL and T6BS.6HL. The high-resolution of GBS read coverage analysis confirmed the results obtained from the molecular marker analysis and cytogenetic methods and detected the presence of a putative pericentric inversion in the T6HS.6BL and T6BS.6HL lines. Stable centric fusion lines with good yield potential can serve as promising genetic materials for chromosome-mediated improvement of mineral components in wheat, which are in increasingly demand as the global population grows.

## Supplementary Information

Below is the link to the electronic supplementary material.

Supplementary Material 1

Supplementary Material 2

Supplementary Material 3

Supplementary Material 4

Supplementary Material 5

## Data Availability

The datasets generated during the current study are available as supplementary files and from the corresponding author on reasonable request.
