# Supplementary material for: Introgression of barley chromosome arms 4H and 6H into wheat via Robertsonian translocations: GBS-assisted structural analysis and impact on grain nutrient composition
Source: Plant Mol Biol. 2026 Feb 18;116(2):23. doi: 10.1007/s11103-026-01689-8 (PMC12916549; doi:10.1007/s11103-026-01689-8)
Supplement: Supplementary file 5 — Supplementary Material 5 [file 11103_2026_1689_MOESM5_ESM.docx]

**Supplementary material**

**Journal name: Plant Molecular Biology**

**Introgression of barley chromosome arms 4H and 6H into wheat via Robertsonian translocations: GBS-assisted structural analysis and impact on grain nutrient composition**

László Ivanizs^1a^, Eszter Gaál^1a^*, Klaudia Kruppa^1a^, András Farkas^1^, Péter Mikó^1^, Edina Türkösi^1^, Marianna Rakszegi^1^, Péter Kovács^1^, Balázs Kalapos^1^, Andrea Gulyás^1^, Norbert Hídvégi^1^, Kitti Szőke-Pázsi^1^, Márta Molnár-Láng^1^, Éva Szakács^1^, Mahmoud Said^2,3^, Jan Bartoš^2^, Tünde Pusztahelyi^4^, Dimitar Douchkov^5^, István Molnár^1^

^1^Agricultural Institute, Centre for Agricultural Research, Martonvásár, Hungary

^2^ Institute for Experimental Botany, Centre of Plant Structural and Functional Genomics, Olomouc 779 00, Czech Republic

^3^ Field Crops Research Institute, Agricultural Research Centre, Giza, Egypt

^4^ Central Laboratory of Agricultural and Food Products, Faculty of Agricultural and Food Sciences and Environmental Management, University of Debrecen, Debrecen, Hungary

^5^ Research Group Biotrophy and Immunity, Leibniz Institute of Plant Genetics and Crop Plant Research (IPK), Gatersleben, Germany

^a^These authors are equal first authors

*Corresponding author: gaal.eszter@atk.hun-ren.hu

Following supplementary informations are involved in these files:

Four tables are included separately in four additional Excel files-

**Supplementary Table 1.** Data on primer pairs used to detect barley chromosome arms.

**Supplementary Table 2.** Results of selection for the plants carrying T6BS.6HL or T6HS.6BL.

**Supplementary Table 3.** Results of selection for the plants carrying T4BS.4HL.

**Supplementary Table 4.** Number of reads, number of average reads per 1Mb bin and average of normalized read values per 1 Mb bin data of Asakaze wheat, Manas barley and T6HS.6BL, T6BS.6HL and T4BS.4HL lines mapped onto the *T. aestivum*-*H. vulgare* *in silico* hybrid.

Ten inline figures are inserted in this file-

**Supplementary Figure S1.** Identification of chromosomes of the euploid T6BS.6HL (centric fusion) line (F_3_ generation) in four different cells (a-d) using fluorescence *in situ* hybridization.

**Supplementary Figure S2.** Identification of chromosomes of the euploid T6HS.6BL (centric fusion) line (F_3_ generation) in four different cells (a-d) using fluorescence *in situ* hybridization.

**Supplementary Figure S3.** Identification of chromosomes of the euploid T4BS.4HL (centric fusion) line (F_3_ generation) in two different cells (a and b) using fluorescence *in situ* hybridization.

**Supplementary Figure S4.** Digital capillary electrophoretic pattern of molecular markers specific to barley chromosome 4H.

**Supplementary Figure S5.** Normalized GBS read coverage of the Asakaze parental wheat variety along the 1-7 chromosomes of wheat subgenomes A, B, and D, and the barley genome H used as reference.

**Supplementary Figure S6.** Normalized GBS read coverage of the Manas parental barley variety along the 1-7 chromosomes of wheat subgenomes A, B, and D, and the barley genome H used as reference.

**Supplementary Figure S7.** Normalized GBS read coverage of the T6HS.6BL line (F_5_ generation) along the 1-7 chromosomes of wheat subgenomes A, B, and D, and the barley genome H used as reference.

**Supplementary Figure S8.** Normalized GBS read coverage of the T6BS.6HL line (F_5_ generation) along the 1-7 chromosomes of wheat subgenomes A, B, and D, and the barley genome H used as reference.

**Supplementary Figure S9.** Normalized GBS read coverage of the T4BS.4HL line (F_5_ generation) along the 1-7 chromosomes of wheat subgenomes A, B, and D, and the barley genome H used as reference.

**Supplementary Figure S10.** Spike morphology of the wheat cultivars Rannaja and Asakaze, the barley cultivar Manas and the wheat-barley translocation lines.

**
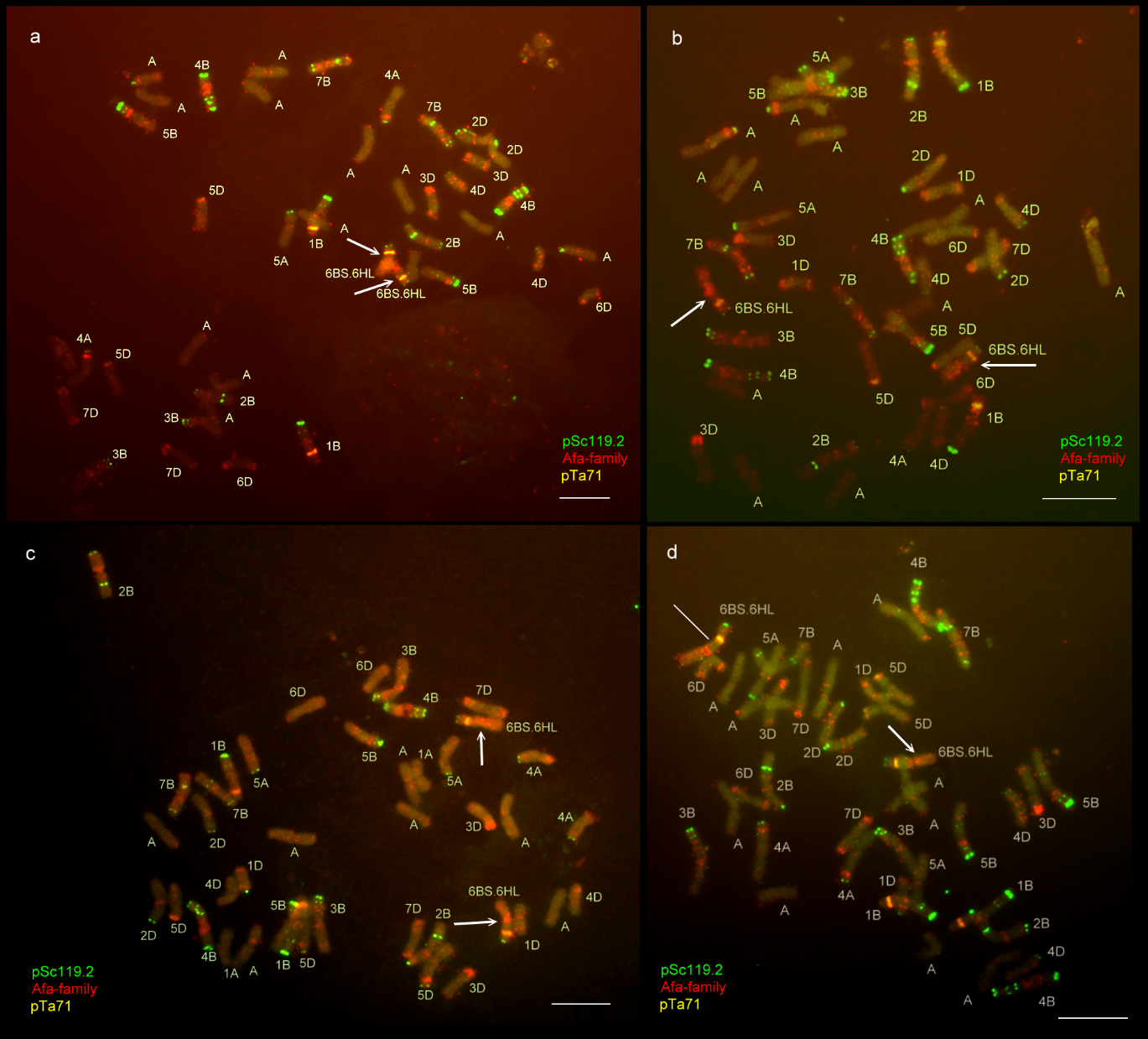
**

**Supplementary Figure S1.** Identification of chromosomes of the euploid T6BS.6HL (centric fusion) line (F_3_ generation) in four different cells (a-d) using fluorescence *in situ* hybridization. Wheat-barley centric fusion chromosomes are indicated by arrows. Scale bar = 10 μm.

**
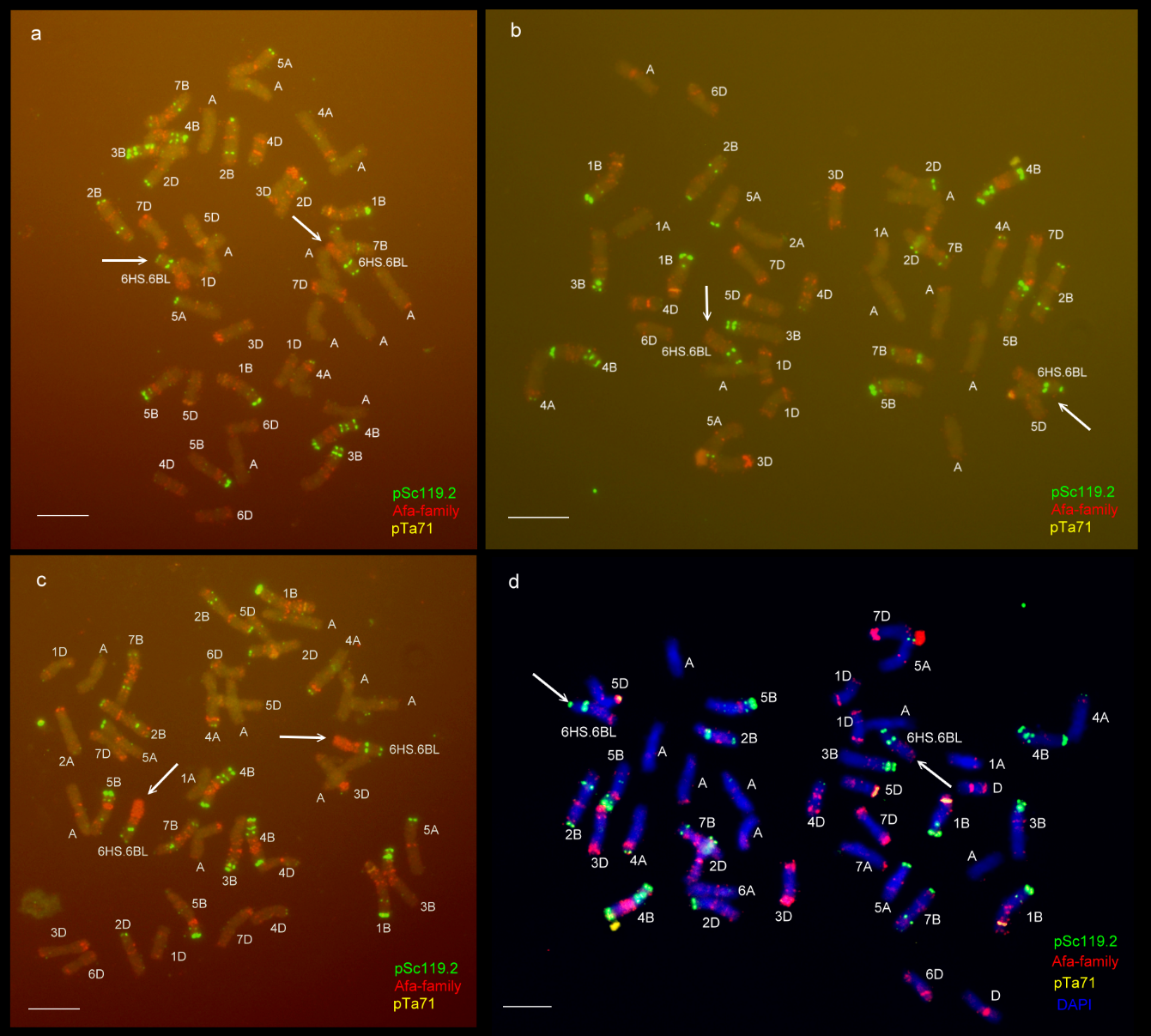
**

**Supplementary Figure S2.** Identification of chromosomes of the euploid T6HS.6BL (centric fusion) line (F_3_ generation) in four different cells (a-d) using fluorescence *in situ* hybridization. Wheat-barley centric fusion chromosomes are indicated by arrows. Scale bar = 10 μm.


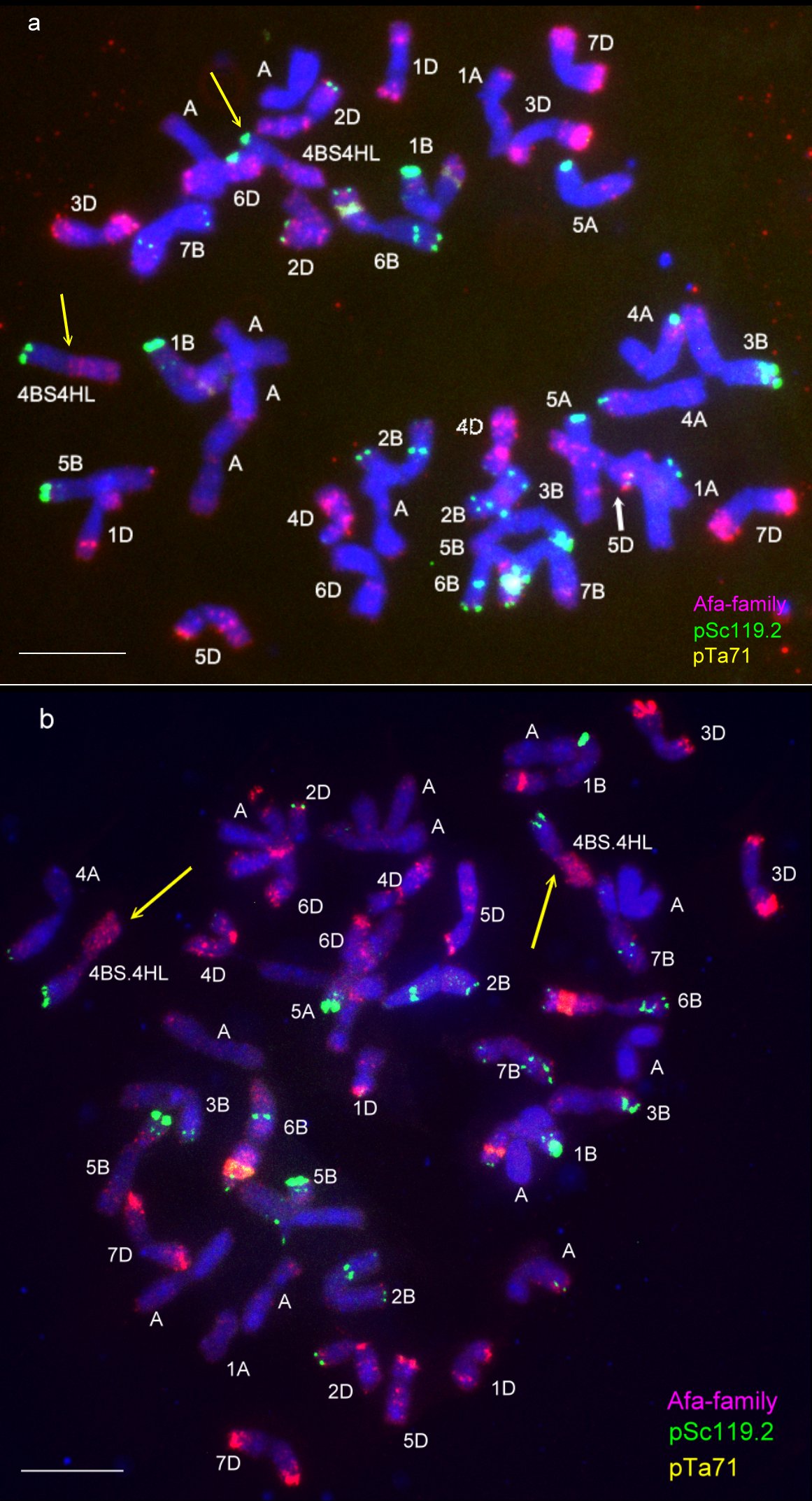


**Supplementary Figure S3.** Identification of chromosomes of the euploid T4BS.4HL (centric fusion) line (F_3_ generation) in two different cells (a and b) using fluorescence *in situ* hybridization. Wheat-barley centric fusion chromosomes are indicated by arrows. Scale bar = 10 μm.


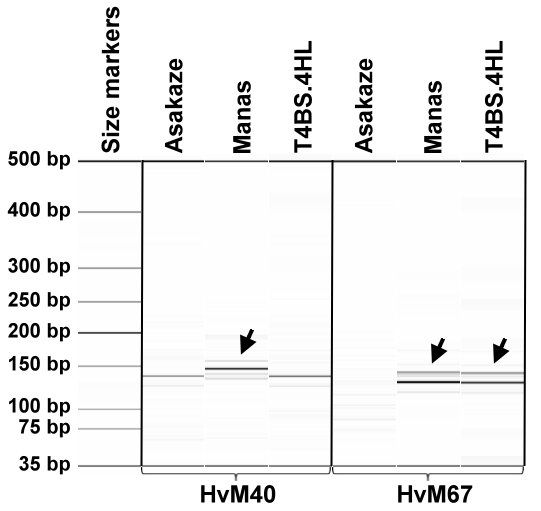


**Supplementary Figure S4.** Digital capillary electrophoretic pattern of molecular markers specific to barley chromosome 4H. The *HvM40* marker, located on the short arm (4HS), and the *HvM67* marker, mapped to the long arm (4HL) of chromosome 4H, were tested on wheat cultivar Asakaze, and barley cultivar Manas and line T4BS.4HL/Asakaze. The arrow indicates the presence of barley-specific PCR products in the tested samples. A 35-500 bp DNA ladder was used as a molecular-weight size standard to estimate the fragment size.


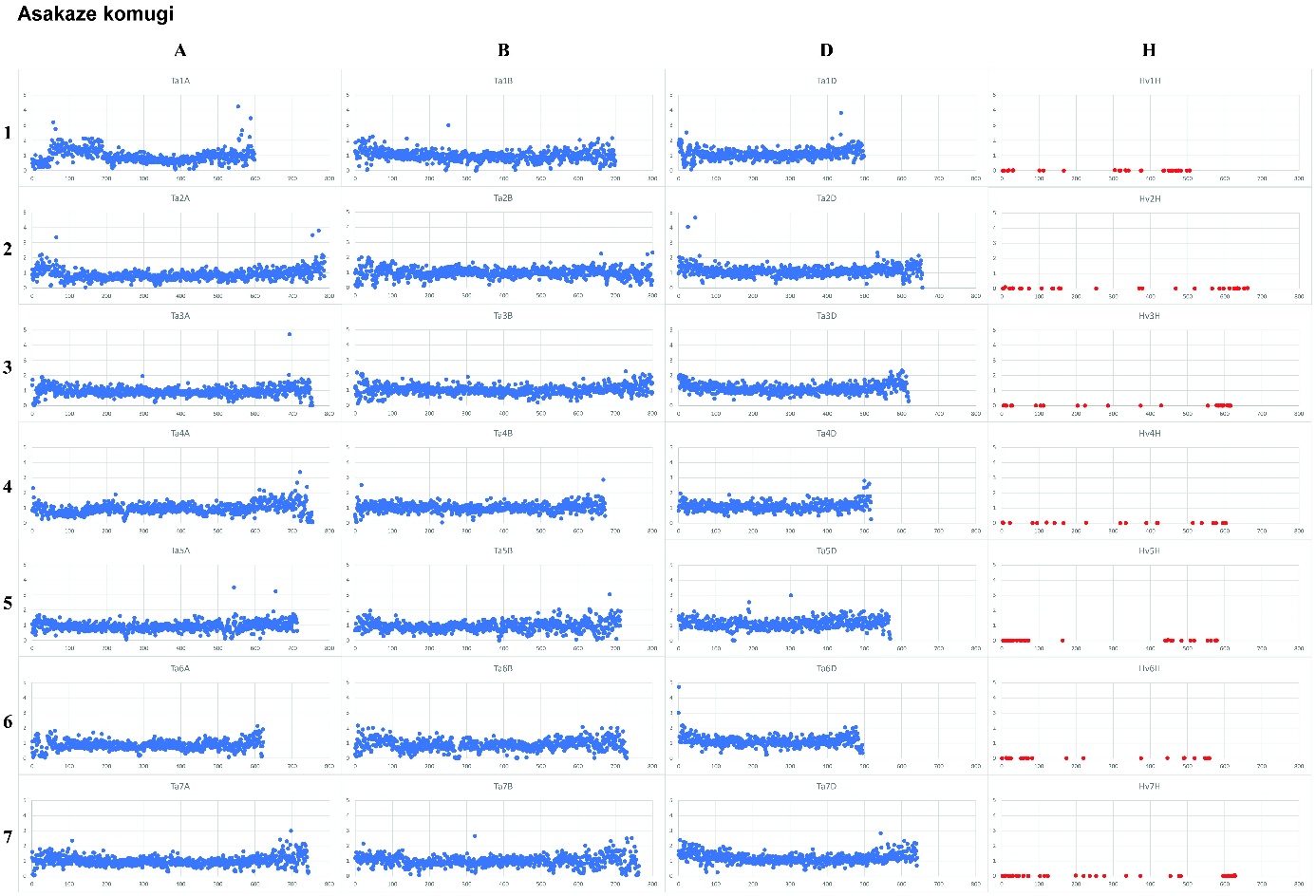


**Supplementary Figure S5.** Normalized GBS read coverage of the Asakaze parental wheat variety along the 1-7 chromosomes of wheat subgenomes A, B, and D, and the barley genome H used as reference. The x-axis represents the genomic position within the chromosome in Mb, while the y-axis shows the normalized read coverage values.


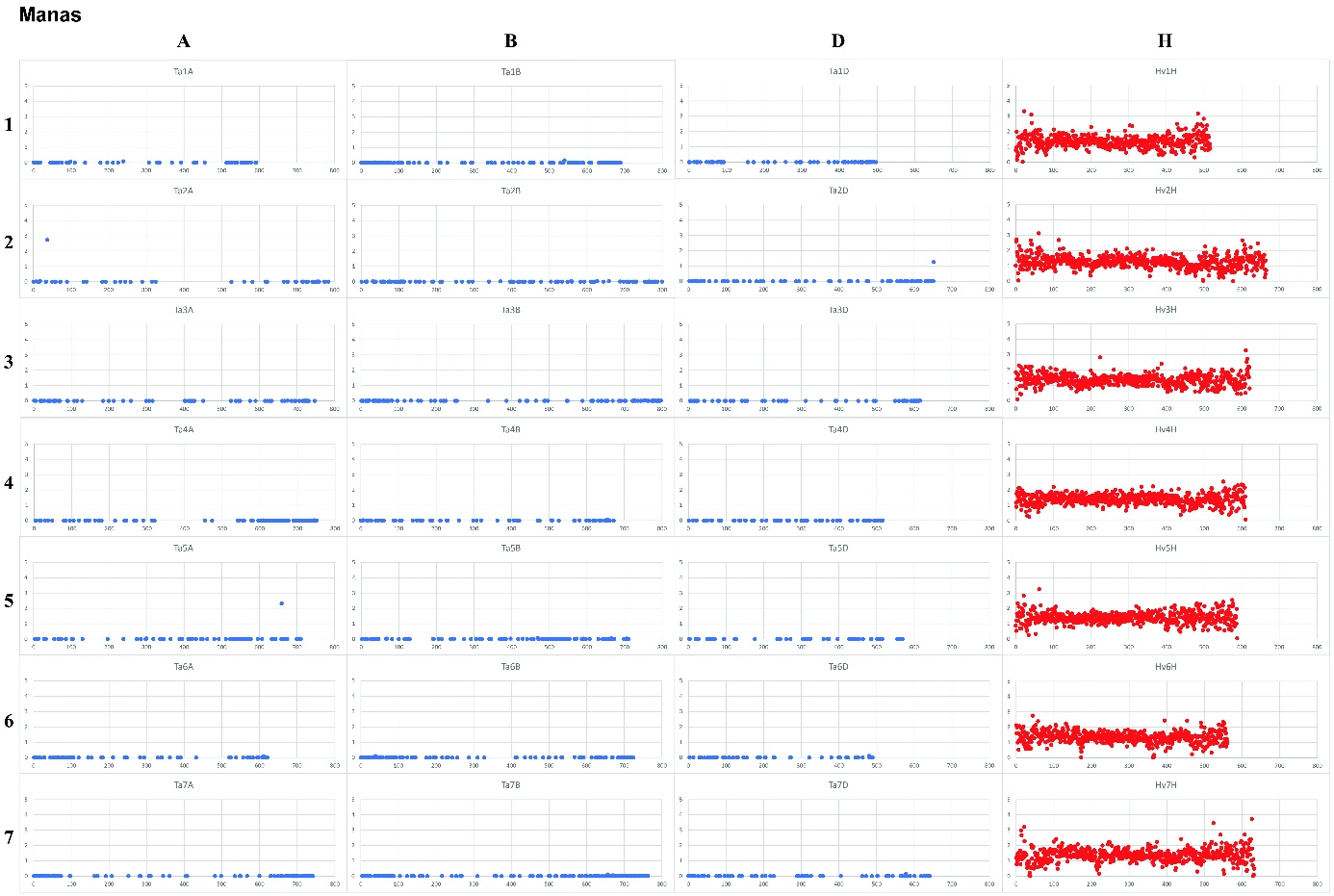


**Supplementary Figure S6.** Normalized GBS read coverage of the Manas parental barley variety along the 1-7 chromosomes of wheat subgenomes A, B, and D, and the barley genome H used as reference. The x-axis represents the genomic position within the chromosome in Mb, while the y-axis shows the normalized read coverage values.


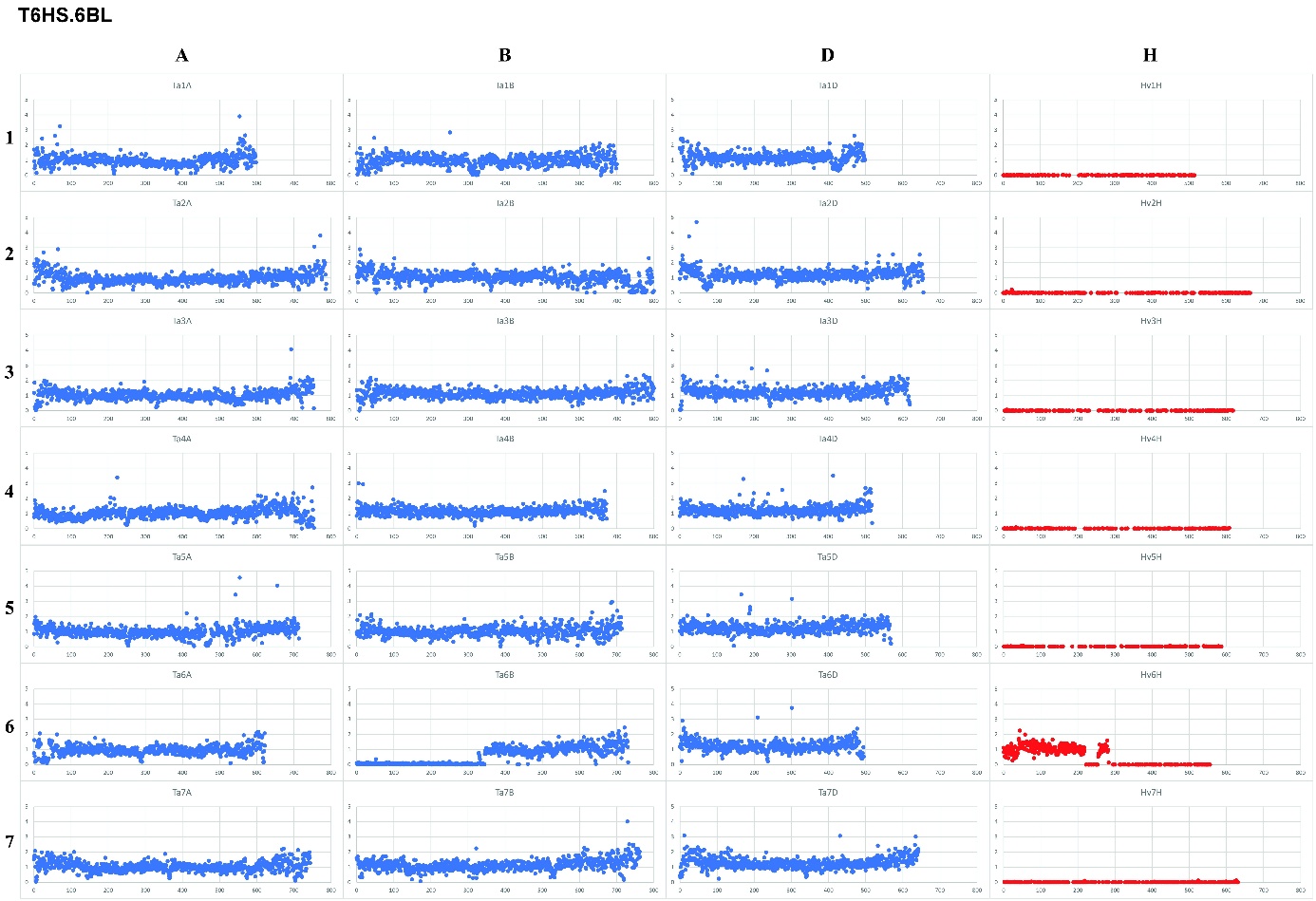


**Supplementary Figure S7.** Normalized GBS read coverage of the T6HS.6BL line (F_5_ generation) along the 1-7 chromosomes of wheat subgenomes A, B, and D, and the barley genome H used as reference. The x-axis represents the genomic position within the chromosome in Mb, while the y-axis shows the normalized read coverage values.


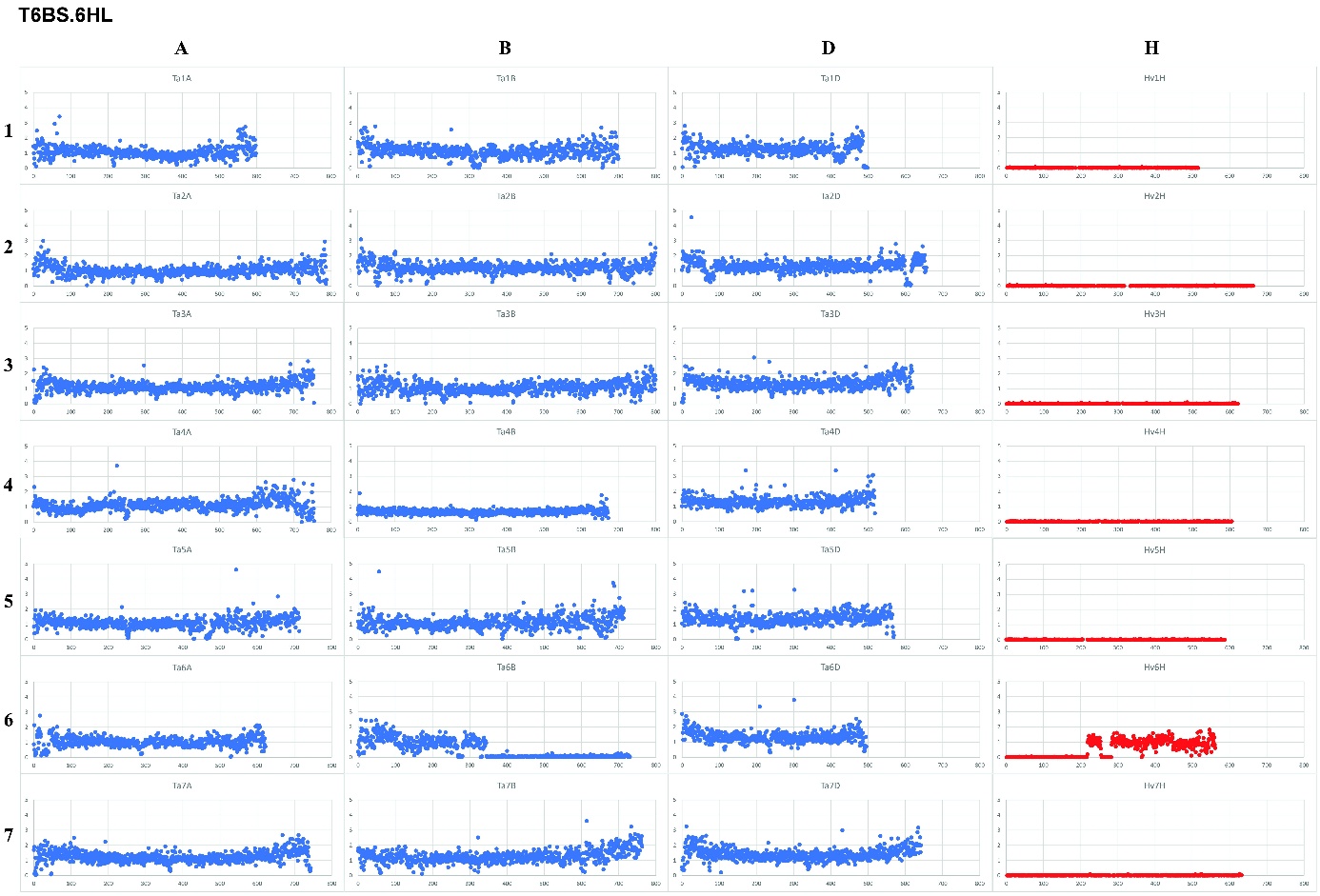


**Supplementary Figure S8.** Normalized GBS read coverage of the T6BS.6HL line (F_5_ generation) along the 1-7 chromosomes of wheat subgenomes A, B, and D, and the barley genome H used as reference. The x-axis represents the genomic position within the chromosome in Mb, while the y-axis shows the normalized read coverage values.


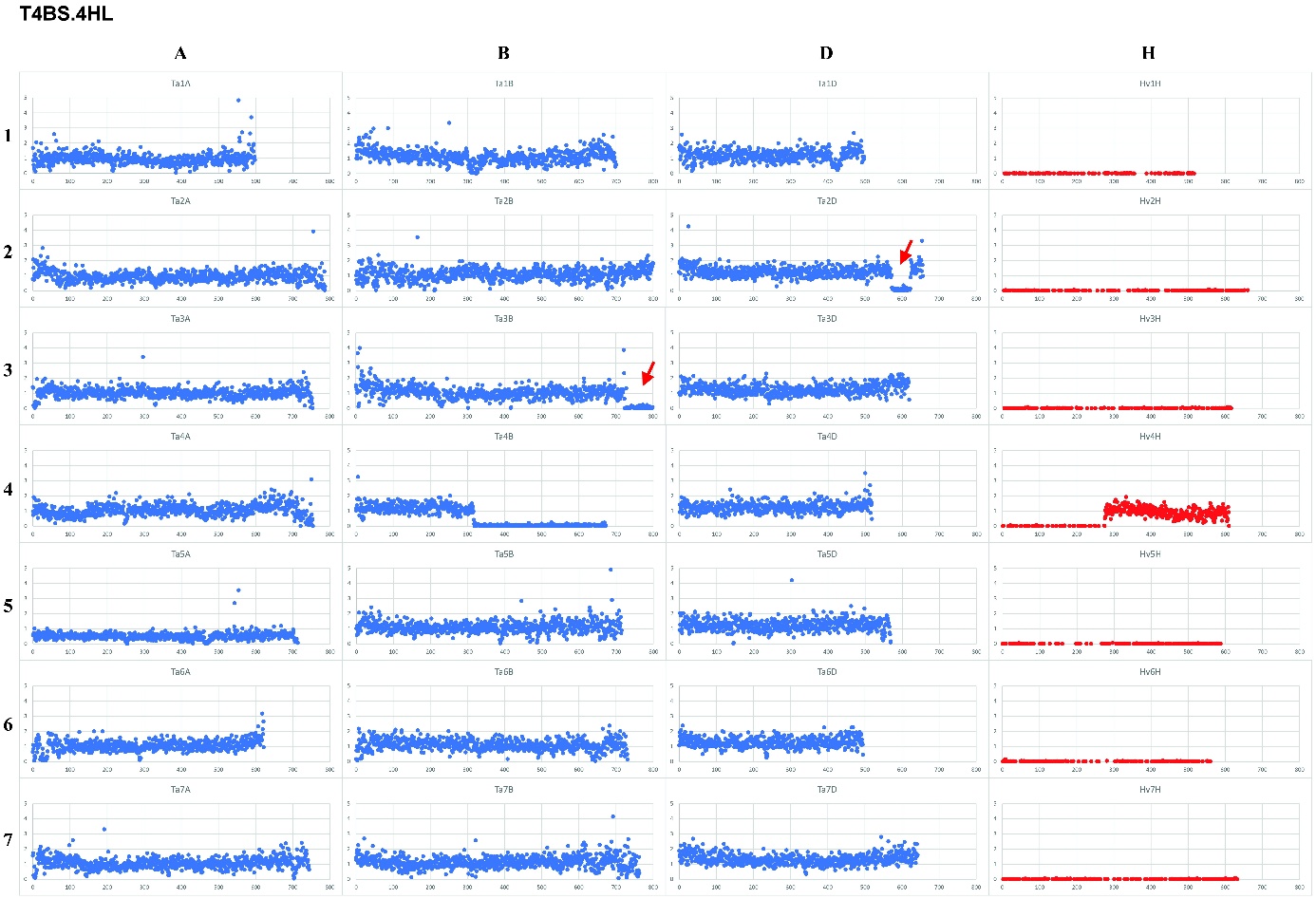


**Supplementary Figure S9.** Normalized GBS read coverage of the T4BS.4HL line (F_5_ generation) along the 1-7 chromosomes of wheat subgenomes A, B, and D, and the barley genome H used as reference. The x-axis represents the genomic position within the chromosome in Mb, while the y-axis shows the normalized read coverage values. Red arrows indicate the two regions (49 and 127 Mb) that are missing from wheat chromosomes 2D (573-622 Mb) and 3B (724-851 Mb).


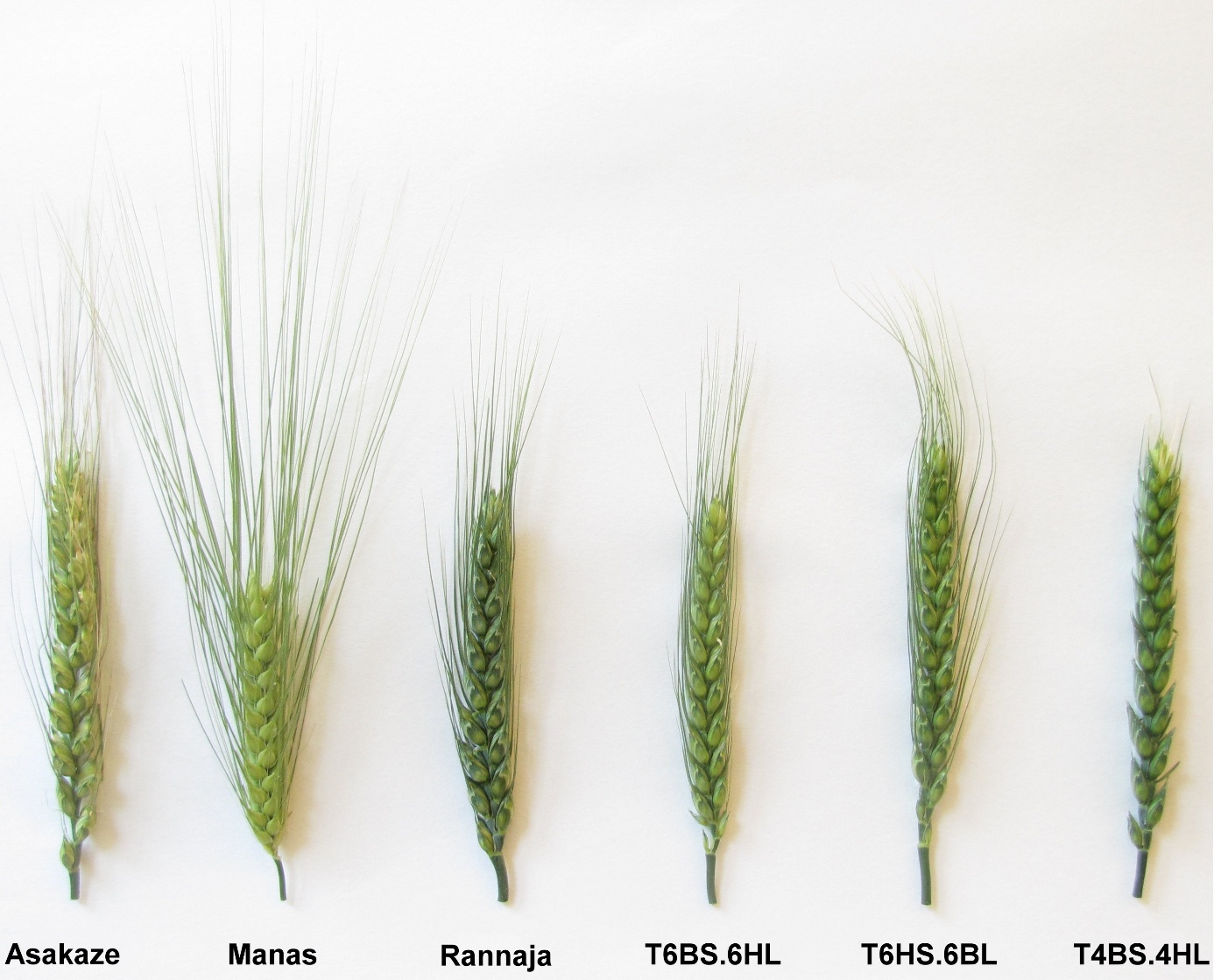


**Supplementary Figure S10.** Spike morphology of the wheat cultivars Rannaja and Asakaze, the barley cultivar Manas and the wheat-barley translocation lines. The plants were grown in the Organic Nursery (Tükrös) in Martonvásár, Hungary in the 2023-24 growing season.
